# Fibrolytic Bacteria Isolated from the Rumen of North American Moose (*Alces alces*) and Their Use as a Probiotic in Neonatal Lambs

**DOI:** 10.1371/journal.pone.0144804

**Published:** 2015-12-30

**Authors:** Suzanne L. Ishaq, Christina J. Kim, Doug Reis, André-Denis G. Wright

**Affiliations:** 1 Department of Animal Science, College of Agriculture and Life Sciences, University of Vermont, Burlington, Vermont, United States of America; 2 Department of Microbiology and Molecular Genetics, College of Agriculture and Life Sciences, University of Vermont, Burlington, Vermont, United States of America; University of Wisconsin-Madison, UNITED STATES

## Abstract

Fibrolytic bacteria were isolated from the rumen of North American moose (*Alces alces*), which eat a high-fiber diet of woody browse. It was hypothesized that fibrolytic bacteria isolated from the moose rumen could be used as probiotics to improve fiber degradation and animal production. Thirty-one isolates (*Bacillus*, n = 26; *Paenibacillus*, n = 1; and *Staphylococcus*, n = 4) were cultured from moose rumen digesta samples collected in Vermont. Using Sanger sequencing of the 16S rRNA gene, culturing techniques, and optical densities, isolates were identified and screened for biochemical properties important to plant carbohydrate degradation. Five isolates were selected as candidates for use as a probiotic, which was administered daily to neonate lambs for 9 weeks. It was hypothesized that regular administration of a probiotic to improve fibrolysis to neonate animals through weaning would increase the developing rumen bacterial diversity, increase animal production, and allow for long-term colonization of the probiotic species. Neither weight gain nor wool quality was improved in lambs given a probiotic, however, dietary efficiency was increased as evidenced by the reduced feed intake (and rearing costs) without a loss to weight gain. Experimental lambs had a lower acetate to propionate ratio than control lambs, which was previously shown to indicate increased dietary efficiency. Fibrolytic bacteria made up the majority of sequences, mainly *Prevotella*, *Butyrivibrio*, and *Ruminococcus*. While protozoal densities increased over time and were stable, methanogen densities varied greatly in the first six months of life for lambs. This is likely due to the changing diet and bacterial populations in the developing rumen.

## Introduction

The North American moose (*Alces alces*) is a large cervid, which consumes a high-fiber diet of woody browse: mainly willow, pine, maple, and fir [1, 2]. They also consume seasonally available aquatic vegetation, which is higher in sodium than arboreal vegetation [1]. This diet provides several nutritional challenges for which the moose has adapted, such as tannins [3] and other plant secondary metabolites [2, 4–6]. Few studies have identified the rumen bacteria of moose [7–9], or used culturing techniques to isolate bacteria from the rumen of moose [9]. Previously, it was shown that moose from Vermont contained a higher proportion of bacteria belonging to the phylum Firmicutes, which are mostly fibrolytic [8].

Neonatal ruminants undergo rumen development over a period of 8–12 weeks, during which the rumen and reticulum increase in size and functionality [10]. This process is enhanced by microbial colonization of the rumen and the introduction of a fiber-based diet [11–13]. Initially, lactic acid bacteria (LAB), such as *Streptococcus thermophilus*, *Lactobacillus acidophilus*, or *Bifidobacterium bifidus*, tend to dominate, as well as *Escherichia coli* [14, 15]. While cellulolytic bacteria do appear in the rumen within the first few days of life [14–16], it is not until weaning and a transition to a plant-based diet that they become the dominant type of rumen bacteria [8, 17]. As the microbial diversity adapts to the rumen environment and the diet provided, so too do the gastrointestinal tract epithelia adapt to the microbiota [18, 19]. Thus, introducing new microbiota after these host-microbiota interactions have been made may not be successful.

Rumen development in young animals can be further improved using probiotics [10, 20, 21]. Probiotics for livestock are generally comprised of LAB or fibrolytic bacteria. LAB probiotics are more common in pre-weaned ruminants [22–24] or monogastrics [22, 25], but are also used in adult ruminants [26, 27]. Fibrolytic probiotics have more often been used to improve digestive function in adult ruminants [28, 29], as well as for pre-weaned ruminants [21]. Many studies report short-term beneficial effects only, either due to the production animals reaching market weight, or because the probiotic failed to colonize the digestive tract long-term.

Moose were chosen as the source for probiotic strains as they are highly likely to host efficient species or strains of bacteria, which can digest cellulose, hemicellulose, and lignin. Moose subsist on a diet of woody browse, which is very high in fiber [30–32]. Additionally, their body temperature [33] and dry matter intake (DMI) [34] is more similar to lambs [35] than to calves or goat kids, thus improving the likelihood of survival and long-term rumen colonization by the species of interest.

The present study investigated different species of *Bacillus*, a strain of *Paenibacillus woosongensis*, and several strains of *Staphylococcus saprophyticus*. The industrial applications of *B*. *licheniformis* are extensive due to the breadth of its enzymatic capabilities [36–38], but also because many are thermophilic or halophilic [38,39]. *Paenibacillus woosongensis* was originally isolated from forest soil, and was shown to digest a variety of carbohydrates, including cellulose and xylan [40]. *Staphylococcus saprophyticus* is a cellulolytic bacterium originally isolated from the termite gut [41], which is a fibrolytic environment generally devoid of cellulolytic protozoa.

The ability of rumen microorganisms to degrade multiple plant polysaccharides makes them more competitive in the rumen, as well as makes available more energy from feed for both microorganisms and the host. The ability to survive under restrictive nutritional conditions is an especially important trait for bacteria used in industrial applications, but can also provide an advantage over competitive species or strains of bacteria which require vitamins or other substrates in the rumen. Additionally, bacteria which can positively impact the host would be beneficial to overall animal health in addition to increasing dietary efficiency. Indole production often takes place in the intestines, and is used as a quorum-sensing signal molecule between gut bacteria. However, its presence in the intestines also stimulates cellular junction-associated molecules in gut epithelial cells, and promotes resistance to dextran sodium sulfate (DSS)-induced colitis [42].

It was hypothesized that fibrolytic bacteria from the rumen of moose would be capable of digesting a wide variety of complex carbohydrates, surviving in a wide range of growth parameters, and would make potential candidates for a probiotic in ruminants. It was also hypothesized that regular administration of a probiotic with fibrolytic properties to neonate animals through weaning would increase the developing rumen bacterial diversity, increase animal production, and allow for long-term colonization of the probiotic species. The objectives of this study were to isolate fibrolytic bacteria from the rumen of moose, characterize these isolates, and assess them for their potential as a probiotic for ruminants by evaluating animal growth performance and ruminal parameters.

## Materials and Methods

### Bacterial culturing

Fresh rumen samples were collected during the October, 2010 hunting season in Vermont, with permission of licensed hunters through the Vermont Department of Fish and Wildlife (F&W). No permit is necessary to collect samples from animals legally taken during the hunting season once the kill has been reported to F&W. Moose were shot in the field, and samples were collected during field dressing according to instructions on collecting rumen samples from the middle of the rumen, and minimizing exposure to oxygen. As moose were deceased at time of sampling, collection procedures did not need F&W approval. Samples were put on ice within 2 h of death, and were transferred to the laboratory within 24 h, where they were mixed with an equal volume of 80% glycerol and stored at -80°C until culturing. Additional information regarding the hosts can be found in Ishaq & Wright [7]. Isolates were given unique identifiers (i.e. VTM3R11) containing the following abbreviations: Vermont (VT), moose (M), individual number (1–4), and rumen (R), as well as isolate number.

Bacteria were isolated on M8 agar plates [43], with an added 2 g/L of cellulose and cellobiose, inside an anaerobic chamber (COY Laboratories, Michigan, US). Whole rumen contents were serial diluted in M8 broth, and all dilutions (10^−1^ to 10^−9^) were plated with five replicates. Plates were monitored for up to 7 d, and colonies were picked and re-isolated on fresh media until colonies were shown to be pure using gram staining and colony morphology measurements. A total of 31 isolates were cultured from four individual moose rumen samples, and stock aliquots of each isolate were mixed with 80% glycerol and stored at -80°C. Isolates were tested for their catalase reaction [39].

Monocultures were identified using automated cycle sequencing at the University of Vermont DNA Analysis Facility. The bacterial 16S rRNA gene was amplified using the universal bacterial primers 27F and 1494R [44]. PCR was performed using whole cells from cultures and using the iTaq DNA Polymerase kit (Bio-Rad, California, US) following the manufacturer’s instructions. PCR conditions were: initial denaturation of 94°C for 5 min, then 33 cycles of 94°C for 30 s, 50°C for 30 s, and 72°C for 1 min, followed by a final extension of 72°C for 6 min. PCR was performed on a C1000 thermal cycler (Bio-Rad, California, US). Recalcitrant isolates were first extracted using the DNA extraction protocol in the QIAamp DNA Stool Mini Kit (QIAGEN, Maryland, USA). Sequences were proofread using ChromasPro ver. 1.7.5, aligned using the CLUSTALW algorithm in MEGA ver. 6.0, and then used to calculate pairwise genetic distance using the Kimura 2-parameter model [45]. Sequences were classified using BLAST (NCBI), and a neighbor joining tree was generated using MEGA.

As cellulose in the broth media prevented accurate optical density measurements, isolates were subcultured into 10 ml of tryptic soy broth (TSB) (1% vol/vol inoculation), and then incubated for 24 h at various temperatures or pH (adjusted prior to autoclaving). Optical density was used to determine relative growth using a Spectronic 200 (ThermoScientific, California, US), with absorbance measured at 600 nm [46]. All samples were run in triplicate, and optimal ranges were set as all isolates measuring >0.5% absorbance. Optimal salinity was measured as growth on tryptic soy agar (TSA) media containing 4–15% NaCl. Heat tolerance was tested by immersing 48 h old cultures in a 60°C water bath for 30 min, then inoculating TSA plates (1% vol/vol inoculation) and incubating at 37°C for 72 h to observe for growth. Isolates which were able to survive >55°C were tested for their ability to tolerate sodium azide. Isolates were grown on azide dextrose media (tryptone, 15 g/L; beef extract, 4.5 g/L; glucose, 7.5 g/L; sodium chloride, 7.5 g/L; and sodium azide, 0.2 g/L; pH 7.2), incubated at 45°C for 5 d, and observed for growth.

Isolates were tested for their ability to digest complex carbohydrates (cellulose, cellobiose, carboxymethylcellulose, xylan, and starch) or plant components (lignin) on minimal media containing only one carbon source and salts [47]. Minimal media plates were incubated at 37°C for up to 2 wk to observe for growth. Isolates were tested on mannitol media for their ability to metabolize mannitol and tolerate potassium tellurite. To test for the production of the aromatic compound indole from the amino acid tryptophan, isolates were grown in 1% w/v tryptone broth for 14 d, after which Kovac’s reagent was added to the culture broth to test for a color reaction [48]. Isolates were subcultured into Simmon’s Citrate slants [49] and Propionate slants [39] for 7 d, and observed for bacterial growth and color change to indicate the ability to use citrate or propionate, respectively, as a carbon source and ammonia as a nitrogen source [50]. To test the ability to reduce nitrate to nitrite, isolates were subcultured into nitrate broth for 2 and 7 d and then tested for color change using potassium iodine strips moistened with 1N HCl [51].

Six isolates (VTM2R66, VTM1R74, VTM2R84, VTM4R85, VTM1R92, and VTM1R96), which were able to survive at a wide range of temperature, pH, salinity, and nutritional conditions, were selected for further investigation into whether the isolates would be good candidates for use as a probiotic for ruminants. In addition to being able to grow under a wide range of conditions, all six isolates were facultative anaerobes, making them more likely to survive the process by which they were mixed and administered to lambs. As per Food and Drug Administration (FDA) regulations, probiotics must maintain 10^7^ CFUs for the duration of its shelf life. Isolates were cultured separately in M8+cellulose broth biweekly for approximately six months to determine whether the isolate could be maintained for an extended period at sufficient concentrations to be used as a probiotic. Concentration was measured by number of colony forming units (CFUs)/ml on a plate count, performed in duplicate. The five isolates were then tested for their ability to survive in commercial milk replacer for up to 72 hr at 37°C, and maintain a minimum density of 10^7^. Isolates were cultured for 24, 48 and 72 hr in DuMOR Blue Ribbon lamb milk replacer (Tractor Supply Co, Vermont, USA), reconstituted according to manufacturer’s instructions, and then replated on M8+cellulose for plate counts at 24 h. Purity was determined via weekly gram staining, and occasional Sanger sequencing.

### Lamb probiotic trial experimental design

All animal procedures were approved by the University of Vermont (UVM) Institutional Animal Care and Use Committee (IACUC protocol 14–008). This portion of the project was funded by a Graduate Fellowship from the USDA National Institute of Food and Agriculture’s Agriculture and Food Research Initiative (Grant # 2013-67011-21152). Results are presented by date and/or experimental period (week). Twenty Dorset-cross lambs, 4–7 d of age, were purchased from Bonnieview Farm (Craftsbury, VT). Lambs were group housed at the Miller Research Farm at UVM (Burlington VT), beginning on April 22, 2014. Eighteen male and two female lambs were randomly assigned to either the control (n = 10) or the experimental (n = 10) group with nine males and one female per group. Males were castrated within the first two weeks of the study, and groups had similar weights (mean 5.9 ± 0.2 kg) prior to the beginning of the study. Water was provided ad libitum.

For four weeks, lambs were fed DuMOR lamb milk replacer above recommended servings (Tractor Supply Co, Vermont, USA) using bucket feeding systems (Premier 1 Supplies, Iowa, USA), and group intake was recorded. Any remaining milk replacer was measured and subtracted from intake once lambs had finished feeding and moved away from feeding buckets. Beginning in week five, lambs were also given DuMOR sheep starter pelleted grain feed (Crude Protein (min.) 16.00%, Crude Fat (min.) 2.00%, Crude Fiber (max.) 16.00%) based on recommended servings (Tractor Supply Co, Vermont, USA), and group intakes were recorded. Any remaining grain was measured and subtracted from intake. At the end of week five, lambs were weaned off of the milk replacer and were fed grain pellets and timothy hay, and again group intake was recorded with wasted hay being subtracted from intake. Lambs were considered fully weaned at 9 weeks when they were transferred to pasture and were no longer receiving milk replacer or lamb starter grain. At experimental week 9 (June 26, 2014), when lambs were 9.5–10 weeks old, they were transferred to Sterling College (Craftsbury, VT), where they were maintained as a single mob grazing on pasture until mid-October, 2014.

### Probiotic

Five bacterial isolates were chosen for use as a probiotic as follows, with GenBank accessions numbers in parentheses: *Bacillus foraminis* VTM4R85 (KP245773), *B*. *firmus* VTM2R84 (KP245774), *B*. *licheniformis* VTM2R66 (KP245781), *B*. *licheniformis* VTM1R74 (KP245789), and *Staphylococcus saprophyticus bovis* VTM1R96 (KP245800). Isolates were selected based on their ability to digest carboxymethylcellulose, cellobiose, cellulose, lignin, starch, and xylan on minimal media. Isolates were also able to survive at a wide range of temperatures, salinities, and pH.

Isolates were grown individually in M8+cellulose broth, checked regularly for purity using gram staining, and concentrations were measured using standard plate counts. Twenty-four hour old cultures were combined at equal concentration within 1 h prior to administration and kept on ice during transport. Final individual concentration was per FDA probiotic regulations, 10^7^ CFUs/ml. One ml of inoculant or blank media was administered orally via 3 ml syringe to experimental and control lambs, respectively, daily between noon and 1 pm. After two weeks, when lambs were approximately 20 d old, the dose was increased to 2 ml/day. Probiotic or blank media was given daily for 9 weeks until four weeks after weaning at 9.5 to 10 weeks of age.

### Production

Lambs were weighed weekly until probiotic was no longer given, and lambs were put on pasture, then weight was monthly for the duration of the study. At the end of study week 9, when lambs were put on pasture, a 2 x 2 in patch was shaved on the side, within 3–5 in of the spine (i.e. mid-side sample). Wool was allowed to grow out for 14 weeks, after which a 1 x 1 in patch was shaved, dried, weighed, and sent for fiber testing to Yocom-McColl Testing Labs in Denver, CO. Significance for this and other statistics was calculated using Student’s T-test, and deviation is presented as standard error mean (SEM).

### Rumen sampling

Rumen samples were collected weekly for eight weeks, and then monthly once lambs were on pasture. Samples were collected in the morning, roughly from 9–11 am. Sample collection was within one to two hours of feeding, approximately 20 h after the last probiotic dose (given from noon to 1 pm daily). While on pasture, when no probiotic was administered, samples were collected from 9–11 am. In both cases, esophageal tubing was used to obtain samples directly from the rumen, from which up to 15 ml of fluid and particulate matter were collected and put on ice immediately until transfer back to the lab. Some rumen fluid (approximately 7 ml) was separated out and used to measure pH and volatile fatty acids (VFA). Rumen pH was tested using a MW101 pH meter (Milwaukee, North Carolina, USA). VFAs and ethanol were measured using gas chromatography at the William H. Miner Agricultural Research Institute (Chazy, NY). Thawed rumen samples were centrifuged for 20 min at 4° at 10,000 x G. Supernatant was filtered through a single layer of Whatman filter paper, and 0.8 ml of filtrate was mixed with an equal volume of internal standards (oxalic acid and trimethylamine). Significance for this and other measurements was calculated using Student’s T-test, and deviation is presented as standard error mean (SEM).

### Sequencing and DNA data analysis

For consistency in DNA extraction, all samples were frozen for two weeks before processing, as not all samples were able to be processed immediately. DNA was extracted from individual samples using the QIAamp DNA stool fast kit (QIAGEN, MD), and the V1-V3 region of the 16S rRNA gene was amplified using previously described protocols [8]. Amplicons were sent to Molecular Research DNA (MR DNA) in Shallowater, TX for sequencing with Illumina MiSeq ver. 3. All sequence data is available from the Sequence Read Archive (SRA) under Bioproject PRJNA281251.

Sequences were analyzed using MOTHUR ver. 1.31 [52, 53]. Sequences were trimmed to remove barcodes and primers, as well as any sequence that contained a mismatch in the barcode, more than two mismatches in the primer, sequences with homopolymers >8, sequences <475 bases or >570 bases, and sequences with an average quality score <32 over 5 bases. Sequences were aligned to the Silva 16S rRNA bacteria MOTHUR reference file, which had been modified to include moose fibrolytic isolates cultured in the laboratory, including the five which were used in the probiotic. The reference alignment was also trimmed to begin at 27F and end after 800 bases. Chimeras were identified using UCHIME [54] and removed. Sequences were identified using the k-nearest neighbor method. Data were subsampled to 10,000 sequences per sample, clustered with a 0.03% genetic cutoff using the nearest neighbor method into Operational Taxonomic Units (OTUs), which at this cutoff are species-level groupings for statistical comparison. The following diversity parameters were measured and presented as group mean: ACE [55], CHAO [56], Good’s Coverage [57], Shannon-Weiner diversity [58], Inverse Simpson [59], Analysis of Molecular Variance (AMOVA), and UniFrac values [60].

In order to compare control and experimental groups from all four time points, sequences which passed quality analysis were pooled, and were subsampled to 2,000 sequences per sample, giving 20,000 per group per time point. This subsample was used to create a neighbor-joining tree using the mothur-integrated algorithms for Clearcut [61], linear discriminant analysis (LDA) using the mothur-integrated Lefse [62], and principal coordinate analysis (PCoA) [63].

### Real-time PCR

Real-time PCR was used to calculate archaeal and protozoal densities in whole samples. DNA was amplified using a CFX96 Real-Time System (Bio-Rad, CA) and a C1000 ThermalCycler (Bio-Rad, CA). Data were analyzed using CFX Manager Software ver. 1.6 (Bio-Rad, CA), and are presented as corrected rRNA copy number/ml. The iQ SYBR Green Supermix kit (Bio-Rad, CA) was used: 12.5 μl of mix, 2.5 μl of each primer (40mM), 6.5 μl of ddH_2_O, and 1 μl of the initial DNA extract diluted to approximately 10 ng/μL. For methanogens, the primers targeted the methyl coenzyme-M reductase A gene (mcrA), following the protocol by Denman et al. [64]. The internal standards for methanogens were a mix of *Methanobrevibacter smithii*, *M*. *gottschalkii*, *M*. *ruminantium* and *M*. *millerae* (R^2^ = 0.998) at five different concentrations, run in triplicate.

For protozoa, the primers, PSSU316F and PSSU539R [65], targeted the 18S rRNA gene, following the protocol by Sylvester et al. [65]. The internal standards for protozoa were created in the laboratory using fresh dairy cattle rumen contents which were filtered through one layer of cheesecloth to remove large particles, and then the protozoa were allowed to separate in a funnel for two hours at 39°C. Once a protozoal pellet was visible, 50 ml were drawn from the bottom of the funnel, and 1 volume of 100% ethanol was added to fix the cells and DNA. The mix was centrifuged for 5 min at 2,000 x G, the pellet was washed with TE buffer (1MTris-HCl, 0.5 M EDTA, pH 8.0), and then centrifuged again. Cells were counted microscopically using a Thoma Slide following the protocol by Dehority [66]. DNA was extracted and used at five different concentrations, run in triplicate (R^2^ = 0.998). Both protocols were followed by a melt curve, with a temperature increase 0.5°C every 10 s from 65°C up to 95°C to check for contamination. Correlation between methanogen and protozoal densities in the lamb rumen was measured by R^2^ values based on linear regression.

## Results

### Isolates

**Fig 1:**
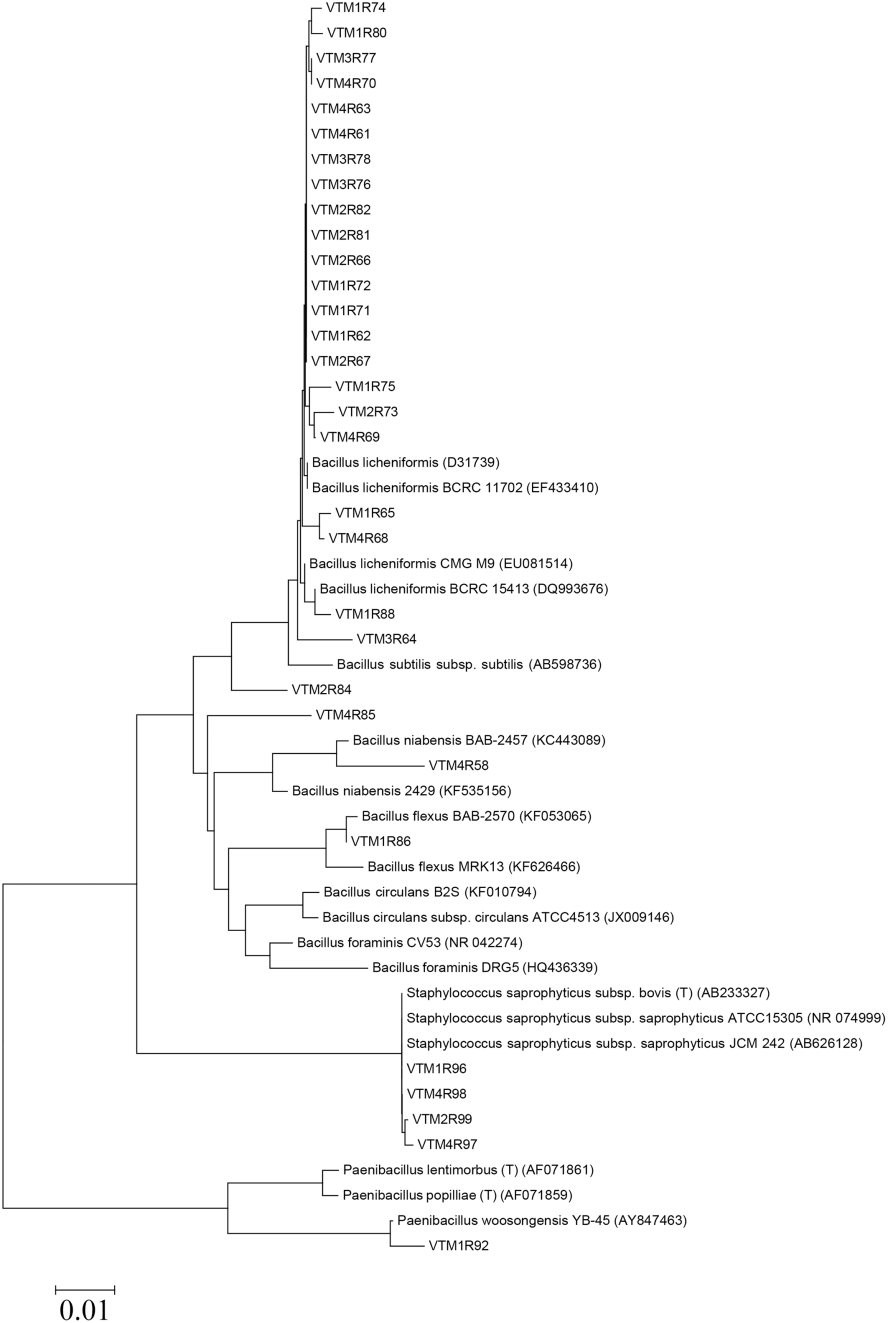
Phylogenetic comparison of 31 fibrolytic isolates along with known sequences (NCBI). A neighbor-joining tree was created using MEGA ver. 6 and the Kimura 2-parameter model. The asterisk (*) indicates the five probiotic isolates, and the one potential probiotic strain which could not be grown in continuous culture.

All 31 isolates were gram positive and catalase positive. Isolates had the following percent identity to known sequences in NCBI: *Bacillus licheniformis*, 98–100% (n = 22); *B*. *foraminis*, 98% (n = 1); *B*. *firmus*, 98% (n = 1); *B*. *flexus*, 100% (n = 1); *B*. *niabensis*, 98% (n = 1); *Paenibacillus woosongensis*, 98% (n = 1); and *Staphylococcus saprophyticus*, 99–100% (n = 4) (Table 1, Fig 1). All 16S rRNA sequences are available from GenBank (NCBI) under the accession numbers KP245773–KP245803.

**Table 1:** Isolate GenBank ID, closest GenBank match with percent identity, growth on minimal media or on high salinity, and ability to reduce nitrate. CM = carboxymethylcellulose, CB = cellobiose, LG = lignin, ST = starch, XY = xylan, 8% = 8% NaCl, 10% = 10% NaCl, NR = nitrate reduction.

Isolate	GenBank ID	Closest GenBank Identification	CM	CB	LG	ST	XY	8%	10%	NT
VTM4R85	KP245773	98% *B*. *foraminis*	+	+	+	+	+	-	-	-
VTM2R84	KP245774	98% *B*. *firmus*	+	+	+	+	+	+	+	+
VTM1R86	KP245775	100% *B*. *flexus*	+	+	-	+	+	+	+	+
VTM4R61	KP245776	99% *B*. *licheniformis*	+	+	+	+	+	+	+	+
VTM1R62	KP245777	99% *B*. *licheniformis*	+	+	+	-	+	+	+	-
VTM4R63	KP245778	99% *B*. *licheniformis*	-	+	-	+	+	+	+	-
VTM3R64	KP245779	99% *B*. *licheniformis*	+	+	+	+	+	+	+	+
VTM1R65	KP245780	98% *B*. *licheniformis*	+	+	+	+	+	+	+	-
VTM2R66	KP245781	99% *B*. *licheniformis*	+	+	+	+	+	+	-	-
VTM2R67	KP245782	100% *B*. *licheniformis*	-	+	-	-	+	-	-	-
VTM4R68	KP245783	98% *B*. *licheniformis*	-	+	+	+	+	+	+	+
VTM4R69	KP245784	99% *B*. *licheniformis*	+	+	+	+	+	+	+	+
VTM4R70	KP245785	99% *B*. *licheniformis*	-	+	-	-	+	-	-	-
VTM1R71	KP245786	99% *B*. *licheniformis*	+	+	-	+	+	+	-	+
VTM1R72	KP245787	99% *B*. *licheniformis*	-	+	-	-	-	+	+	-
VTM2R73	KP245788	99% *B*. *licheniformis*	+	+	-	+	+	-	-	+
VTM1R74	KP245789	99% *B*. *licheniformis*	+	+	+	+	+	+	+	+
VTM1R75	KP245790	99% *B*. *licheniformis*	-	-	-	-	-	-	-	-
VTM3R76	KP245791	99% *B*. *licheniformis*	+	+	+	+	+	+	+	-
VTM3R77	KP245792	99% *B*. *licheniformis*	-	+	+	-	-	-	-	-
VTM3R78	KP245793	99% *B*. *licheniformis*	-	-	+	-	+	-	-	-
VTM1R80	KP245794	99% *B*. *licheniformis*	+	+	-	+	+	-	-	+
VTM2R81	KP245795	99% *B*. *licheniformis*	-	-	-	-	-	-	-	-
VTM2R82	KP245796	99% *B*. *licheniformis*	+	+	+	+	+	+	-	+
VTM1R88	KP245797	99% *B*. *licheniformis*	+	+	-	+	+	-	-	+
VTM4R58	KP245798	98% *B*. *niabensis*	+	+	+	-	+	+	+	-
VTM1R92	KP245799	98% *P*. *woosongensis*	+	+	+	+	+	+	+	+
VTM1R96	KP245800	100% *S*. *saprophyticus bovis*	+	+	+	+	+	+	+	-
VTM4R98	KP245802	100% *S*. *saprophyticus bovis*	+	+	+	+	+	+	+	-
VTM4R97	KP245801	99% *S*. *saprophyticus saprophyticus*	-	+	-	-	-	-	-	-
VTM2R99	KP245803	99% *S*. *saprophyticus saprophyticus*	+	+	-	+	+	-	-	+
Total positive (n = 31)			21	28	18	21	26	19	16	14

B = Bacillus; P = Paenibacillus; S = Staphylococcus

**Fig 2:**
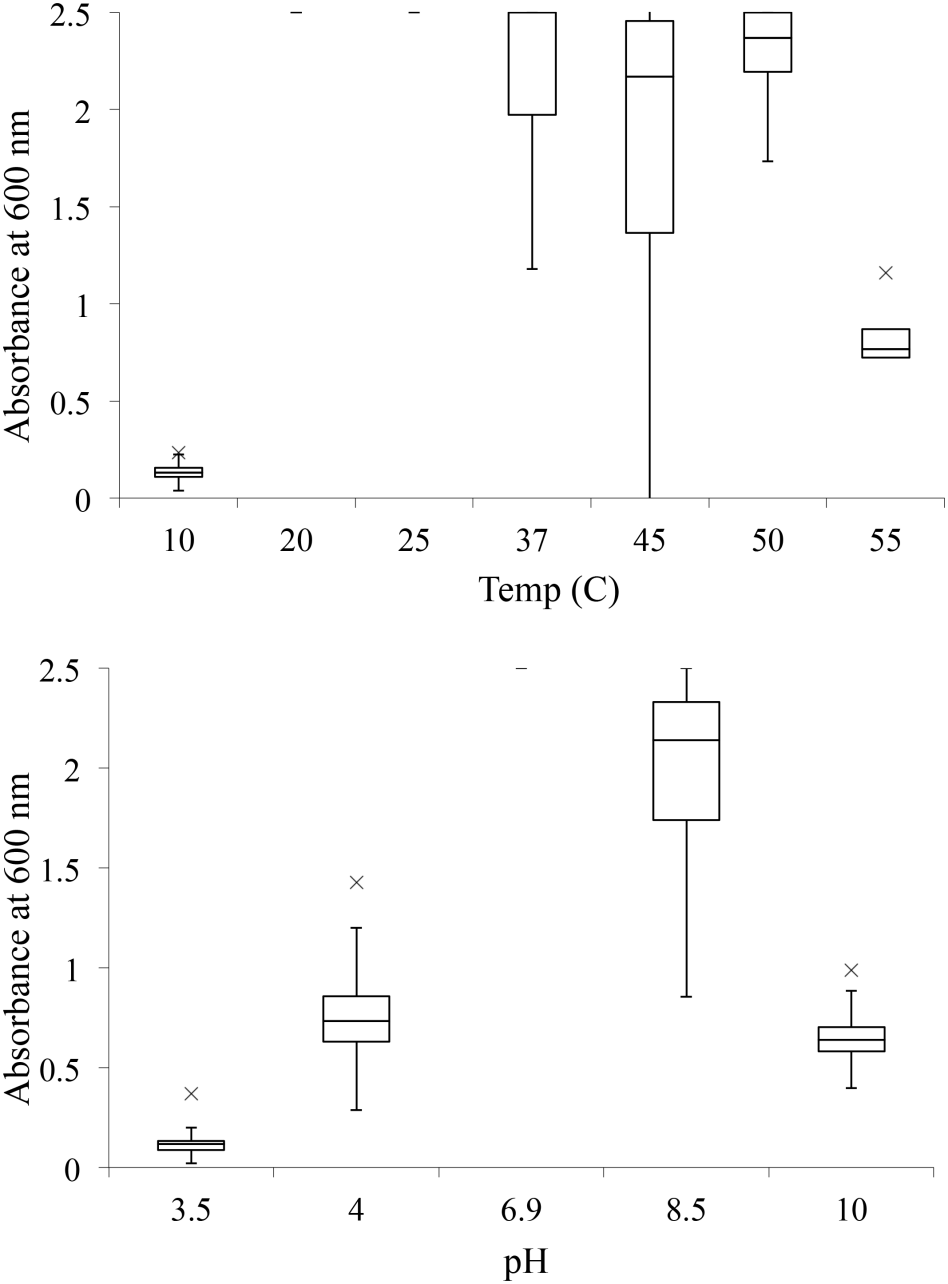
Growth of fibrolytic bacterial isolates at various temperatures (A) and pHs (B), as measured by optical density at 600 nm. The “-” at 20C and 25C indicates all samples were at max absorbance and there was no distribution.

All 31 isolates tolerated 4% NaCl (data not shown). Isolates (n = 16) were able to tolerate up to 10% salinity (Table 1), including *B*. *firmus*, *B*. *flexus*, some *B*. *licheniformis*, *B*. *niabensis*, *P*. *woosongensis*, and some *S*. *saprophyticus*. Isolates from all species grew to >0.5 absorbance between pH 4.0 (n = 27) and pH 10.0 (n = 27), and between 20°C (n = 28) and 55°C (n = 30) (Fig 2). The “-” at 20C and 25C indicates all samples were at max absorbance and there was no distribution. Twenty-nine isolates exhibited normal growth after heat shock, but two isolates (*B*. *niabensis* VTM4R58, and *S*. *saprophyticus* VTM2R99) exhibited no growth. All but one *B*. *licheniformis* isolate (VTM3R64) tolerated sodium azide and exhibited growth after 5 d.

Under minimal conditions, isolates were able to digest cellobiose (n = 28), xylan (n = 26), starch (n = 21), carboxymethylcellulose (n = 21), and lignin (n = 18) (Table 1). All 31 isolates were able to grow on cellulose, glucose, and lactose (data not shown), and 13 isolates were able to digest all four additional carbohydrates as well as lignin (Table 1). Twenty-seven isolates were able to metabolize mannitol, but four *B*. *licheniformis* could not (VTM2R66, VTM1R71, VTM1R80, VTM1R88). Only two *B*. *licheniformis* isolates (VTM2R66, VTM2R82) and one *B*. *foraminis* isolate (VTM4R85) could reduce tellurite. Two *B*. *licheniformis* isolates (VTM1R74, VTM1R75), one *B*. *firmus* isolate (VTM2R84), and one *B*. *foraminis* isolate (VTM4R85) were able to produce indole from tryptophan. All isolates were able to use citrate and propionate as their carbon source, and use ammonia for nitrogen. Twelve isolates were able to reduce nitrate to nitrite after 48 h, and an additional two isolates (*S*. *saprophyticus* VTM2R99 and *B*. *firmus* VTM2R84) were able to reduce nitrate to nitrite after 7 d of growth (Table 1).

### Probiotic

Five isolates (VTM2R66, VTM1R74, VTM2R84, VTM4R85, VTM1R96), which were selected for further testing, maintained concentrations ranging from 10^7^ to 10^10^ CFUs over six months. One isolate (VTM1R92) could not be maintained with sufficient growth about 10^5^ CFUs and was not used in the final probiotic. The same five isolates were able to maintain densities greater than 10^7^ CFUs in liquid lamb replacer over 72 h.

### Production

Total and mean group weight were higher, though not statistically significant (P > 0.05), in the control group, with the exception of week 8 and week 15 (Table 2). Feed intake was lower in experimental lambs for milk replacer and starter grain from week 1–7 (P < 0.001), and while intake was high in experimental lambs for starter grain and timothy hay from week 7–9, this was not significantly different (P = 0.2) (Table 2). The total cost at the end of weaning, when lambs were transferred to pasture (week 9) differed between groups for milk replacer (P = 0.001) and starter grain (P < 0.05) which were lower in the experimental group, and while timothy hay cost was higher in the experimental group, this was not significant (P > 0.05) (Table 2).

**Table 2:** A comparison of weekly total and mean group weights, intake by feed type, and cost of feed per group weight. Group weights were not statistically significant (P value > 0.05) at any time point.

	**Experimental**					
	**Total kg**	**Mean kg**	**Milk replacer (L)**	**Starter gr ain (oz)**	**Timothy hay (flakes)**	**Intake Cost/kg weight**
Day 0	56.95	5.70				
Week 1	75.90	7.59	161.53	0.00	0.00	$2.31
Week 2	102.65	10.27	182.14	0.00	0.00	$1.94
Week 3	124.40	12.44	212.40	0.00	0.00	$1.92
Week 4	161.75	16.18	238.50	104.00	0.00	$1.64
Week 5	166.00	16.60	111.90	512.00	8.00	$0.95
Week 6	176.85	17.69	0.00	439.50	9.00	$0.19
Week 7	195.75	19.58	0.00	720.00	12.50	$0.15
Week 8	218.25	21.83	0.00	1060.00	15.50	$0.66
Week 9	213.45	21.35	0.00	576.00	15.50	$0.41
Week 15	263.30	26.33	0.00	0.00	0.00	$0
Week 23	314.20	31.42	0.00	0.00	0.00	$0
Total Intake			906.465	3411.5	60.5	
Cost			$1,182.65	$68.22	$363.00	
	**Control**					
	**Total kg**	**Mean kg**	**Milk replacer (L)**	**Starter grain (oz)**	**Timothy hay (flakes)**	**Intake Cost/kg weight**
Day 0	61.75	6.18				
Week 1	82.05	8.21	168.85	0.00	0.00	$2.14
Week 2	110.20	11.02	196.44	0.00	0.00	$1.87
Week 3	139.70	13.97	226.90	0.00	0.00	$1.83
Week 4	168.15	16.82	245.80	216.00	0.00	$1.55
Week 5	167.75	16.78	123.85	631.50	4.00	$0.92
Week 6	193.05	19.31	0.00	552.00	8.50	$0.16
Week 7	200.20	20.02	0.00	720.00	5.00	$0.01
Week 8	217.80	21.78	0.00	1044.00	15.50	$0.63
Week 9	215.45	21.55	0.00	576.00	18.00	$0.37
Week 15	260.10	26.01	0.00	0.00	0.00	$0
Week 23	318.70	31.87	0.00	0.00	0.00	$0
Total Intake			961.84	3739.5	51	
Cost			$1,254.90	$74.78	$306.00	

The total cost at the end of weaning was not significantly lower in experimental lambs (P < 0.05) at weaning at week 9. For the experimental group, feeding cost was $1564.63; at a weight of 213.45 kg for the group, this gives a cost of $7.33/kg of body weight. The total cost of the control group was $1592.17, and at a weaning weight of 215.45 kg this yields a cost of $7.38/kg of body weight. When taking into account the total group weights at market weight (aged six months, week 23), the cost/group dropped to $4.80/kg body weight for the experimental group and $5.00/kg body weight for the control group (P < 0.001).

Mid-side sample wool weight was higher (P = 0.02) in the experimental group (mean = 0.83 g, SEM = 0.5) than in the control group (mean = 0.67 g, SEM = 0.07). Mean fiber diameter (MFD) was not significantly different (P = 0.14) between experimental (MFD = 34 μ, SEM = 0.6, SD = 7.4) and control (MFD = 33.1 μ, SEM = 0.6, SD = 7.7) groups. The experimental group did have a significantly (P = 0.04) lower coefficient of variation (CoV = 21.8) than the control group (CoV = 23.4). Although the experimental group had a higher percentage of fibers that were >30 μm (66.8%, SEM = 3.4 experimental, 62.1%, SEM = 2.7 control), this was not statistically significant (P = 0.11).

**Fig 3:**
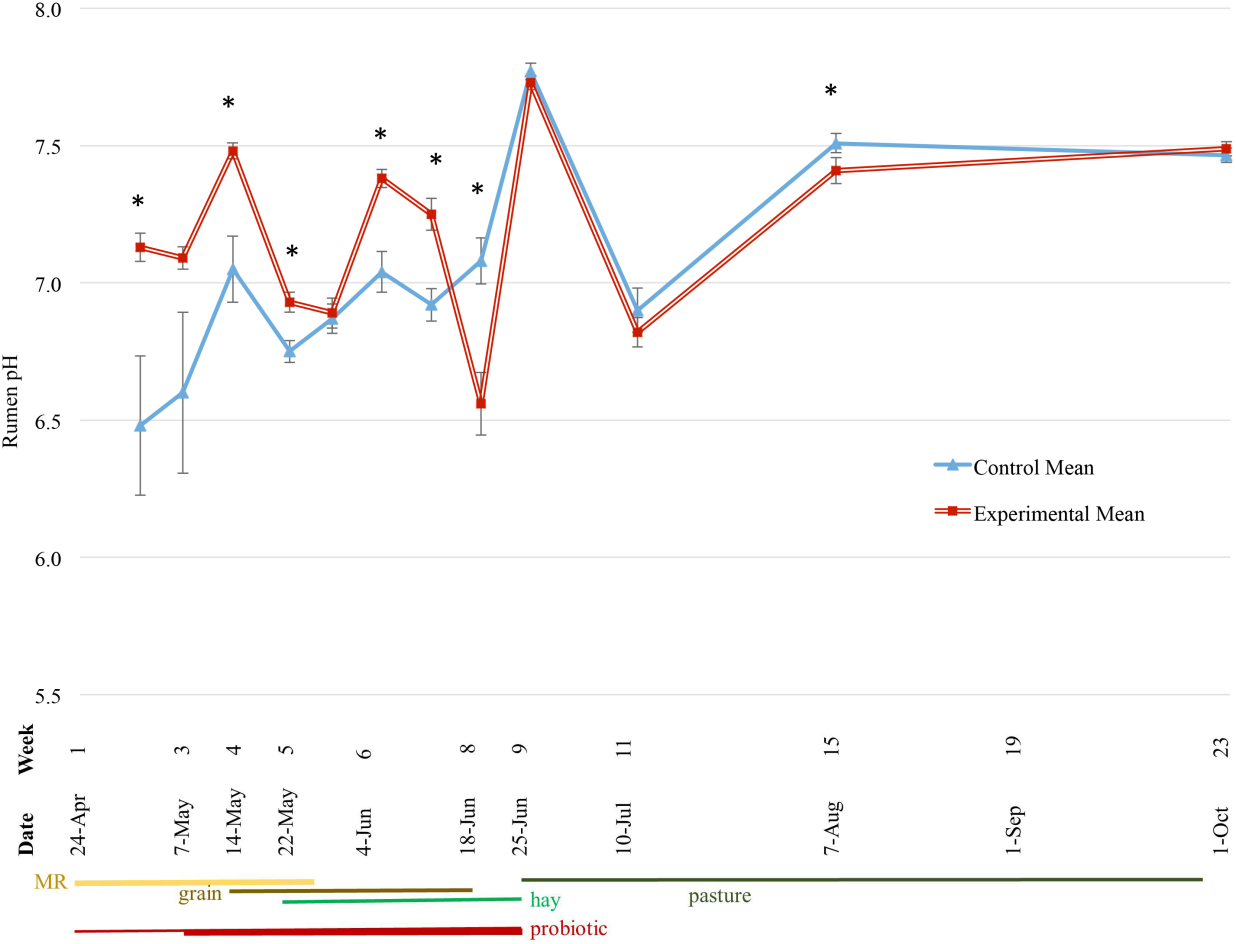
Effect of bacterial probiotic on lamb rumen pH over the first six months of life. Significance (P < 0.05) is denoted with *, and error bars show standard error mean. MR = milk replacer.

The average pH over the course of the experiment was 7.2 for the experimental group and 7.0 for the control group (Fig 3). The experimental group had a higher average pH for the first seven weeks of the experiment and lower variability within the group, while the control group was more likely to have a higher average for the remainder of the study. Seven out of 12 sampling time points were significantly different (Fig 3).

**Fig 4:**
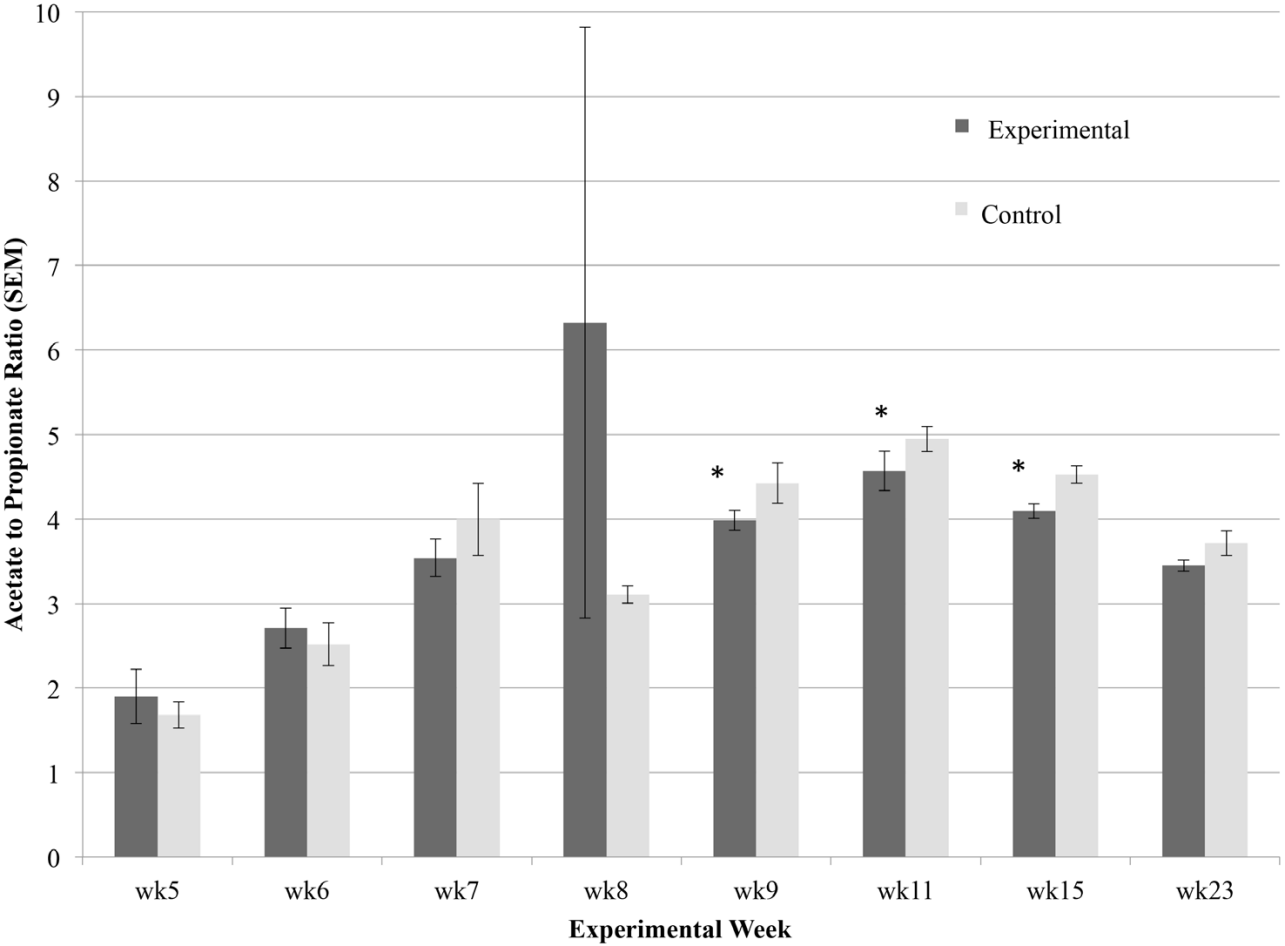
Effect of bacterial probiotic on lamb rumen acetate to propionate ratio over the first six months of life. Significance (P < 0.05) is denoted with *, and error bars show standard error mean.

Total VFAs were significantly different (P < 0.05) in experimental compared to control lambs at weeks 11 and 23 (Table 3). Total VFAs were highest at weeks 8 and 15, and lowest at week 9 after being on a hay-only diet for one week (Table 3). Experimental lambs had statistically higher concentrations than control lambs of butyrate acid in week 5; acetic, butyric, isobutyric, isovaleric, propionate, and valeric acids in week11; lactic and valeric acid in week 15; and acetate, propionate, and butyrate in week 23 (P < 0.05). The acetic acid to propionic acid ratio was statistically lower in the experimental group at weeks 9, 11, and 15 (Fig 4).

**Table 3:** Effect of bacterial probiotic on lamb rumen volatile fatty acid profile and ethanol concentration during the first six months of life. A = acetate, P = propionate, B = butyrate, IB = isobutyrate, IV = isovalerate, L = lactate, V = valerate, EtOH = ethanol.

	A	P	B	IB	IV	L	V	EtOH	A:P	Total (w/ EtOH)	Total (w/o EtOH)
**Week 5**											
Exp	22.79	16.84	5.79	0.11	0.04	1.01	1.25	157.39	1.90	205.22	47.83
Con	23.20	15.28	3.69	0.16	0.05	1.95	0.17	53.42	1.68	97.92	44.50
P	0.46	0.40	0.01	0.03	0.14	0.42	0.20	0.00	0.29	0.01	0.36
**Week 6**											
Exp	26.88	10.70	3.32	0.27	0.13	0.50	0.80	106.93	2.71	149.53	42.60
Con	28.34	12.25	3.01	0.27	0.11	0.62	0.67	71.40	2.52	116.68	45.28
P	0.38	0.22	0.05	0.33	0.47	0.33	0.37	0.29	0.30	0.06	0.35
**Week 7**											
Exp	37.55	11.04	7.94	0.29	0.14	1.50	0.90	1.22	3.54	60.57	59.35
Con	35.86	10.11	5.42	0.37	0.19	4.47	0.67	3.11	4.00	60.21	57.10
P	0.39	0.37	0.02	0.08	0.12	0.11	0.06	0.13	0.18	0.49	0.42
**Week 8**											
Exp	41.33	14.56	8.50	0.42	0.24	4.83	0.91	9.37	6.32	80.17	70.80
Con	41.00	13.28	7.01	0.47	0.24	1.52	0.96	29.41	3.11	93.89	64.47
P	0.48	0.27	0.06	0.15	0.19	0.42	0.16	0.44	0.19	0.16	0.23
**Week 9**											
Exp	15.46	3.92	1.55	0.21	0.08	2.38	0.18	0.37	3.98	24.14	23.78
Con	21.02	4.80	2.32	0.26	0.10	1.79	0.23	0.29	4.43	30.82	30.52
P	0.06	0.14	0.44	0.04	0.08	0.17	0.33	0.11	0.05	0.07	0.06
**Week 11**											
Exp	23.99	5.42	4.44	0.47	0.29	4.21	0.46	1.08	4.57	40.36	39.28
Con	20.65	4.29	2.93	0.36	0.21	3.71	0.28	1.52	4.95	33.96	32.44
P	0.03	0.02	0.37	0.01	0.01	0.01	0.45	0.00	0.03	0.01	0.01
**Week 15**											
Exp	44.26	10.88	6.20	1.00	0.61	1.92	0.50	0.74	4.10	65.91	65.18
Con	48.20	10.76	6.85	1.10	0.67	5.14	0.59	2.15	4.52	75.46	73.31
P	0.23	0.47	0.20	0.23	0.18	0.16	0.02	0.04	0.01	0.04	0.10
**Week 23**											
Exp	38.11	11.14	5.00	0.44	0.26	3.76	0.34	0.25	3.45	59.29	59.04
Con	32.10	8.90	3.96	0.40	0.21	2.02	0.26	0.71	3.71	48.57	47.86
P	0.03	0.04	0.00	0.02	0.15	0.11	0.13	0.08	0.07	0.08	0.03

### Sequencing

**Fig 5:**
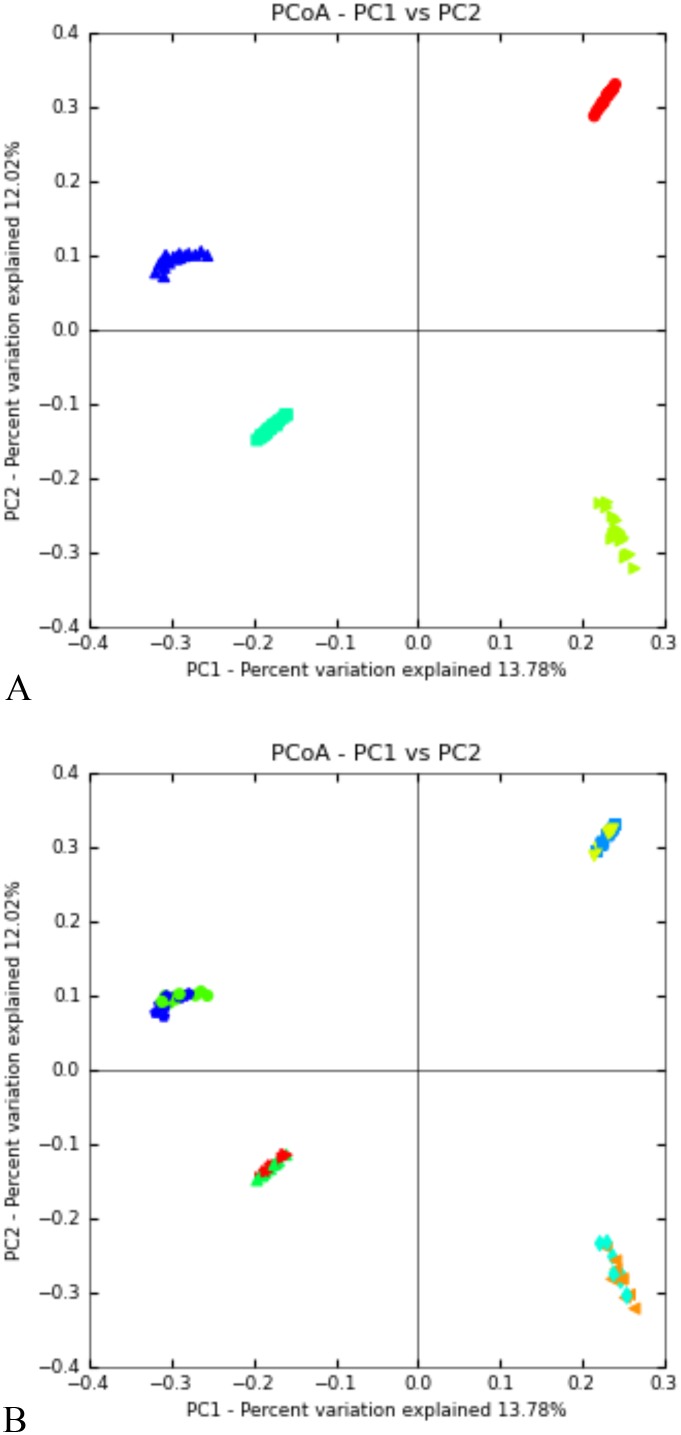
Principal coordinate analysis (PCoA) of lamb rumen bacterial samples by sampling time (A) and treatment (B). Sampling time (A) is week 2 = teal, week 6 = green, week 11 = red, and week 23 = dark blue for both groups. Treatment (B) is control (con) week 2 = red, experimental (exp) week 2 = green triangle, con week 6 = orange, exp week 6 = teal, con week 11 = yellow, exp week 11 = blue, con week 23 = green, exp week 4 = dark blue.

Between 11,000 and 95,000 unique sequences passed quality assurance steps per sample, giving a total of 500,000 to 1.1 million sequences per data set of 20 samples. At the first sampling time point (week 2), after administering the probiotic for a week, groups were statistically different using AMOVA, and unweighted (comparing structure) and weighted (comparing structure and abundance) UniFrac (Table 4). At week 6, CHAO, ACE, Shannon, and Coverage were different between groups, with higher bacterial diversity in the experimental group. Groups were not statistically different on any diversity measure except for unweighted UniFrac at week 11 in July, after being on pasture for two weeks (Table 4). However, by week 23 in October, the control group showed higher diversity (P < 0.05) according to Shannon and Inverse Simpson, and groups clustered separately by weighted and unweighted UniFrac. Principal component analysis (PCoA) graphs showed a strong clustering of groups by sampling time (Fig 5a), but not by treatment (Fig 5b).

**Table 4:** Bacterial diversity statistics per sample for each of the four sampling time points. Results are listed by group, experimental (n = 10) and control (n = 10), or all (n = 20). Using Student’s T-Test: * denotes statistically significant value (P < 0.05) between groups at that time point, and letter superscripts denote statistically significant values between time points for each group. Using two-factor ANOVA with replication: ^1^ denotes statistically significant value for groups at different time points, and ^2^ denotes statistically significant interaction between treatment and time point.

		Sampling dates and experimental week.			
	Group	5-1-14 Wk 2	6-4-14 Wk 6	7-10-14 Wk 11	10-1-14 Wk 23
**Total sequences which passed QA steps**	All	953,581	1,002,520	501,909	1,007,093
**Subsampled to 10,000/sample (200,000/time point)**					
**Good’s Coverage** ^1^	Exp	0.85 ^a^	0.83 * ^a^	0.63 ^b^	0.32 * ^c^
	Con	0.87 ^a^	0.87 ^a^	0.62 ^b^	0.28 ^c^
**Shannon-Weiner Diversity** ^1^	Exp	3.23 ^a^	3.69 * ^a^	5.82 ^b^	7.86 * ^c^
	Con	3.25 ^a^	3.09 ^a^	5.72 ^b^	8.10 ^c^
**Inverse Simpson** ^1^ ^,^ ^2^	Exp	6.34 ^a^	8.02 ^a^	21 ^b^	220 * ^c^
	Con	6.35 ^a^	6.15 ^a^	19 ^b^	447 ^c^
**CHAO** ^1^	Exp	10,907 ^a^	11,489 * ^a^	50,674 ^b^	95,992 ^c^
	Con	8,513 ^a^	7,712 ^a^	57,141 ^b^	111,950 ^c^
**ACE** ^1^	Exp	31,864 ^a^	31,738 * ^a^	149,728 ^b^	278,155 ^c^
	Con	22,578 ^a^	21,378 ^a^	171,878 ^b^	352,438 ^c^
**Total species-level OTUs**	All	20,706	21,035	70,062	106,823
**Mean species-level OTUs/sample** ^1^	Exp	1,271 ^a^	1,425 ^a^	3,918 ^b^	5,700 ^c^
	Con	1,156 ^a^	999 ^a^	3,977 ^b^	6,190 ^c^
**Shared OTUs (sequences)**	All	9 (65,731)	7 (68,258)	20 (64,589)	19 (19,215)
**Group shared OTUs (sequences)**	Exp	13 (72,661)	12 (65,582)	38 (96,060)	31 (78,967)
	Con	12 (77,050)	12 (63,501)	37 (95,910)	33 (81,424)
**AMOVA (p-value)**	All	0.01*	0.73	0.30	0.14
**Weighted UniFrac**	All	0.80 *	0.47	0.44	0.57 *
**Unweighted UniFrac**	All	0.87 *	0.67 *	0.77 *	0.87 *

*Denotes statistically significant value (P < 0.05) between groups at that time point, and letter superscripts denote statistically significant values between time points for each group.

^1^Denotes statistically significant value for groups at different time points, and

^2^denotes statistically significant interaction between treatment and time point.

**Fig 6:**
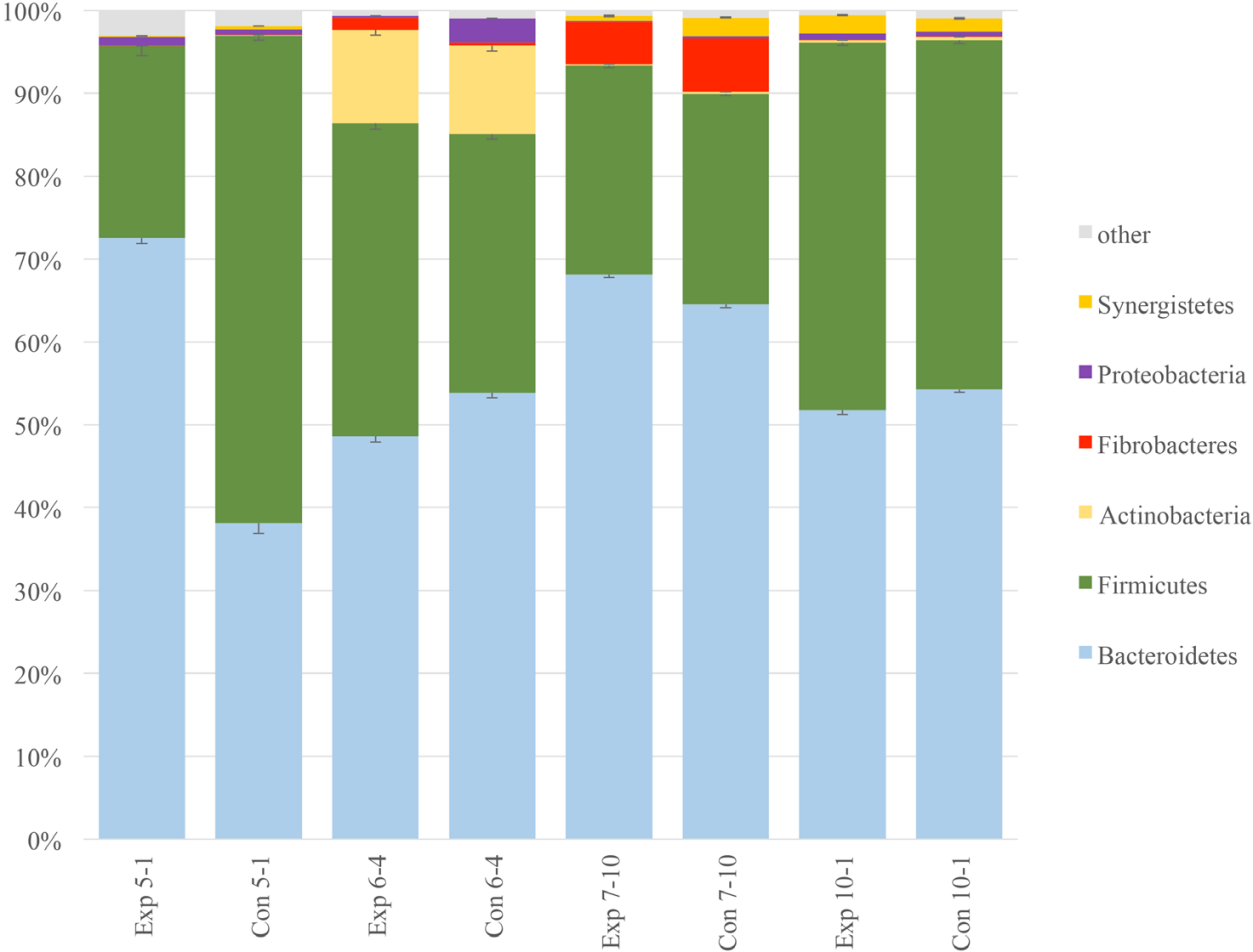
Effect of time and bacterial probiotic on the bacterial diversity at the phylum level of the lamb rumen over the first six months of life. X-axis labeled reflect treatment (Con = control, Exp = probiotic experimental), as well as sampling date. Error bars show standard error mean.

Bacteria belonging to the phylum Bacteroidetes were the most prevalent bacteria (38–73% of total sequences) in both groups for the duration of the study, with the exception of the first sampling of the control group (Fig 6), while bacteria belonging to the phylum Firmicutes were the second most prevalent (23–59%). In the control group, Bacteroidetes bacteria increased while Firmicutes bacteria decreased for the first three samplings (weeks 2, 6 and 11), while the experimental had a general trend of decreasing Bacteroidetes and increasing Firmicutes over time. Other prominent phyla tended to peak at one or two time points, including bacteria belonging to the phyla Actinobacteria and Proteobacteria (week 6), the phylum Fibrobacteres (week 11), and the phylum Synergistetes (weeks 11, 23).

**Fig 7:**
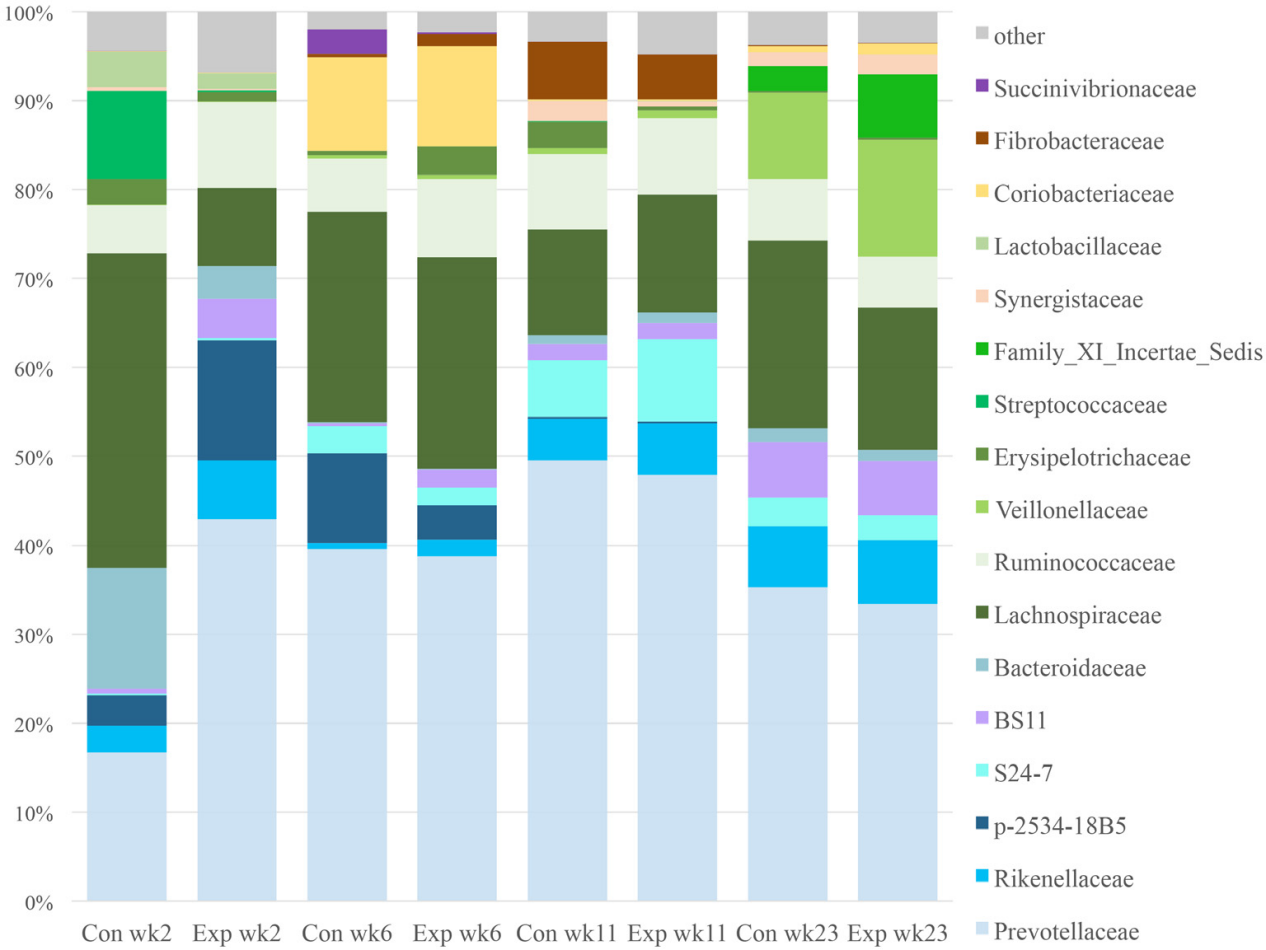
Effect of time and bacterial probiotic on the bacterial diversity at the family level of the lamb rumen over the first six months of life. X-axis labeled reflect treatment (Con = control, Exp = probiotic experimental), as well as sampling date.

Major families identified are shown in Fig 7, with families belonging to Bacteroidetes as shades of blue and members of Firmicutes in shades of green. Prevotellaceae (mostly species *Prevotella*) was a prominent family in all time points and in both groups, but was significantly higher in the experimental group at week 2. Bacteria belonging to the family Lachnospiraceae were also prominent in all samples, although they were significantly higher in the control group at week 2. The experimental group had more Ruminococcaceae bacteria than the control group at weeks 2 and 6. There were also bacterial families that were prominent in only some time points, such as Bacteroidaceae, Streptococcaceae, and the candidate family p-2534-18B5 in week 2 and 6; the Coriobacteriaceae (mostly species *Olsenella*) in week 6, the candidate family S24-7 and Fibrobacteraceae in week 11, Veillonellaceae and the candidate Family XI of the class Bacilli (phylum Firmicutes) in week 23.

While bacteria belonging to the genera *Bacillus* and *Staphylococcus* were found in both groups at all time points, there was not enough resolution in the sequenced amplicons to accurately identify the five probiotic sequences down to species or strain. The total number of all genera identified were as follows: week 2, 301 experimental and 273 control; week 6, 183 experimental and 184 control; week 11, 292 experimental and 331 control; and week 23, 482 experimental and 483 control (S1 Table). Overall, 694 genera were identified across both groups and all time points. The most prevalent genus in all groups and time points was *Prevotella*. Other prominent genera included *Bacteroides*, *Butyrivibrio*, *Catabacter*, *Clostridium*, *Dialister*, *Lactobacillus*, *Olsenella*, *Oribacterium*, *Parvimonas*, *Ruminococcus*, *Selemonas*, *Streptococcus*, and the group RC9 (S1 Table). When comparing all time points together in the smaller subsampled data set, there were 1,787 OTUs identified from the 160,000 sequences, with 88 (4.9%) discriminatory OTUs with LDA >2 (P < 0.05) (Table 5).

**Table 5:** Linear discriminant analysis of bacterial OTUs in experimental and control groups at four sequencing time points.

Taxa	LDA	Taxa	LDA
**Experimental week 2 5-1-14**		**Control week 2 5-1-14**	
Bacteroidales (n = 2)	4.10	*Akkermansia*	3.06
*Fastidiosipila*	2.35	Bacteria	4.55
Neisseriaceae	2.79	Bacteroides	4.60
p-2534-18B5	2.71	*Streptococcus*	4.37
**Experimental week 6 6-4-14**		**Control week 6 6-4-14**	
Clostridiales	3.42	Bacteroidales	3.97
Lachnospiraceae (n = 3)	3.34	Lachnospiraceae	4.57
Ruminococcaceae	3.16	p-2534-18B5	4.82
**Experimental week 11 7-10-14**		*Prevotella*	2.62
*Anaeroplasma*	3.51	Termite_*Treponema*_cluster (n = 2)	2.65
Bacteria	2.58	**Control week 11 7-10-14**	
Bacteroidales (n = 8)	2.84	Bacteria (n = 2)	3.61
*Butyrivibrio-Pseudobutyrivibrio*	2.87	Bacteroidales	2.51
Clostridiales (n = 2)	2.62	Bacteroidetes (n = 2)	2.69
Fibrobacteraceae	2.55	Clostridiales	2.43
Lachnospiraceae (n = 3)	2.95	Lachnospiraceae	2.73
*Oscillospira (n = 2)*	2.67	*Prevotella (n = 2)*	3.24
Planctomycetaceae	2.44	Prevotellaceae	3.34
*Prevotella (n = 2)*	2.75	RC9 (n = 5)	2.80
Prevotellaceae (n = 6)	2.92	Rikenellaceae	2.44
RC9 (n = 7)	2.73	Ruminococcaceae	2.57
Ruminococcaceae	2.99	**Control week 23 10-1-14**	
*Schwartzia*	2.33	Bacteroidales	2.71
**Experimental week 23 10-1-14**		Lachnospiraceae	3.61
Bacteria	2.76	*Prevotella*	2.71
Bacteroidales (n = 2)	2.69	RC9	2.54
BS11 (n = 2)	2.83	Ruminococcaceae	2.51
Prevotellaceae (n = 2)	2.76		
RC9	2.61		
Ruminococcaceae	2.78		

### Real-time PCR

**Fig 8:**
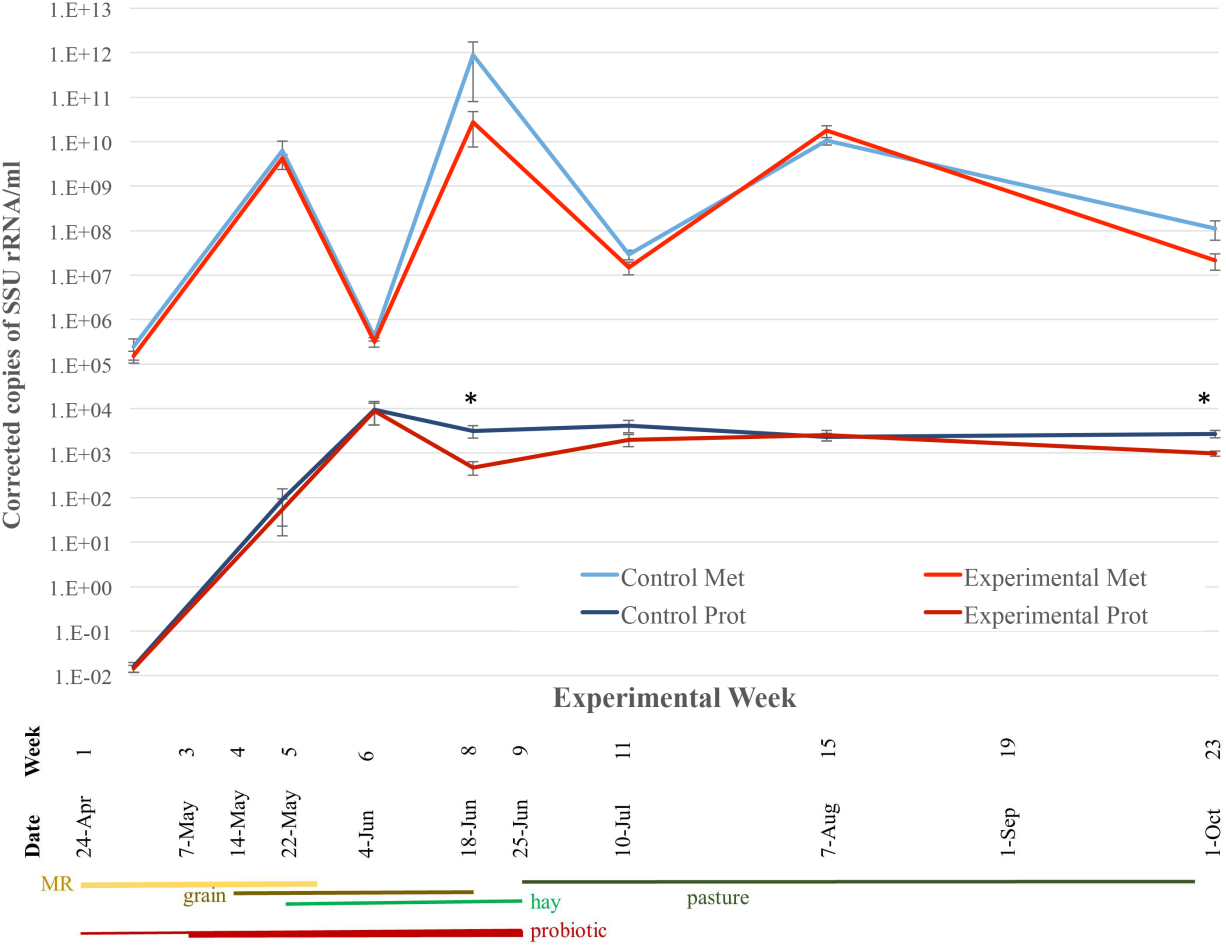
Effect of bacterial probiotic on lamb rumen methanogen and protozoal density, by Real-time PCR. Significance (P < 0.05) is denoted with *, and error bars show standard error mean.

Protozoal density in both control and experimental groups increased over time until it leveled off at approximately 2 x 10^3^ rRNA copes/ml (Fig 8). The control group had statistically higher (P < 0.05) densities at weeks 8 and 23. Methanogen densities increased for the first month, then rapidly decreased at week 6. Levels peaked at week 8, at which point densities decreased to week 11, and then peaked again at week 15. While average density was higher in control lambs for most time points, due to the variability of densities within groups, this was not statistically significant. Overall, protozoal densities were visually positively correlated using linear regression with methanogen densities, though this trend was not statistically significant across all time points (R^2^ = 0.18). However, correlation was high in control lambs at week 1 (R^2^ = 0.88) and week 23 (R^2^ = 0.47), though in experimental lambs the highest was (R^2^ = 0.19) in week 8.

## Discussion

Thirty-one fibrolytic bacterial isolates were examined for their biochemical capabilities and potential as a probiotic for ruminants. Based on their ability to survive a wide range of growth parameters and digest complex carbohydrates on minimal media, many of the 31 fibrolytic isolates in the present study have the potential for use in agricultural or industrial applications. While lambs were not intentionally under restrictive nutritional conditions in this study, bacteria which would still thrive under those conditions if lambs were not eating much of the milk replacer, or were grazing on poor quality pasture would be a better probiotic choice. Additionally, being able to grow up the probiotic species in minimal media would reduce the production costs, as well as ensure that the isolates would be able to withstand potential constraints from an industrial culturing process, if this probiotic were to be commercially produced. However, the ability to survive in the developing digestive tract using milk or milk replacer as a substrate, as well as the ability to consistently grow well in culture, are also important considerations for a viable probiotic product.

The present study tested five isolates as potential probiotics for ruminants. *Bacillus licheniformis* was previously used as a probiotic in dairy cows [67], where it increased milk production and milk protein but not body weight gain. *Bacillus licheniformis* was previously isolated from water buffalo [68] and Native Korean goat [69]. *Bacillus licheniformis* was previously shown to produce bacterocins [68], which can be detrimental to Ruminococcaceae. As *B*. *licheniformis* was not found in large abundance in any sample, and Ruminococcaceae was higher in experimental lambs, this is not likely to be a factor in the present study. *Staphylococcus saprophyticus* was previously isolated from lambs aged two to nine weeks, where their ureolytic ability was noted [70].

Neither weight gain nor wool quality was improved in lambs given a probiotic, however, dietary efficiency was slightly increased as evidenced by the reduced feed intake (and rearing costs) without a significant loss to weight gain. This reduction in rearing costs would be further amplified using more traditional husbandry practices, such as rearing lambs outside and giving them access to grass during weaning, thus precluding the need to supplement with hay. Additionally, the experimental lambs had a lower ratio of acetate to propionate than control lambs, which has previously been shown to indicate increased dietary efficiency [71, 72]. An increased production of propionate reduces free hydrogen in the rumen, making it less available to methanogens. Though not measured here, a reduction in methane production can make more energy available to the host.

While total and individual VFAs, value did not differ significantly between groups, experimental lambs did have higher values for several time points, notably when lambs were on a pasture diet. Acetate, propionate, and valeric acid were increased at several time points, all of which provide energy to the host. Interestingly, very high amounts of ethanol were seen in both groups, but especially the experimental group during the first few VFA sampling time points. Large quantities of ethanol have been observed in the rumen without negative effects to the host, and have acted as a source of energy for the host [73, 74]. Several rumen microorganisms are known to produce ethanol, including *Ruminococcus albus* and some *Clostridium*, although an increase of ethanol has shown mixed results in terms of increasing cellulose digestion and animal production [73]. Previously, *Prevotella* was negatively correlated with isobutyrate and positively correlated with isovalerate [75], yet in the present study, no change in either VFA was seen with relatively high abundance of *Prevotella*.

Sequencing coverage was high in the first two time points, after which it decreased. Conversely, Shannon, Inverse Simpson, CHAO, and ACE indices began low and increased over time, all of which is a function of the increasing diversity of the rumen microbiota as the rumen develops. While there were differences between groups in terms of statistical diversity, there was no difference in OTUs/sample between groups, and this is likely due to evenly subsampling the data set. The experimental group had a higher diversity than the control group at the beginning of the experiment, but this did not persist once the probiotic was no longer administered. Previously, dosing with *Ruminococcus* in adult ruminants was shown to decrease rumen bacterial diversity [29]. Principal component analysis (PCoA) graphs showed a strong clustering of groups by sampling time, but not by treatment, indicating that the change of rumen bacteria over the course of rumen development is a stronger indicator of variance. Additionally, only 25% of the variance was accounted for by these parameters, indicating that other factors, such as individual variation, may account for the high amount of variability in samples.

Fibrolytic bacteria constituted the majority of sequences identified in samples, and while some fibrolytic genera were elevated in the experimental group, this was not consistent across all time points. In all time points and both groups, *Prevotella* was the most prevalent genus, while *Butyrivibrio* and *Ruminococcus* were also prevalent. All three genera have previously been shown to be fibrolytic [76–78]. However, sequences from the probiotic strains could not be confidently reported in the sequenced samples. Previous studies on probiotics have reported similar difficulties identifying probiotics post-dosing [29, 79]. It was thought that using neonate ruminants without a developed microbiota would allow for a small volume and lower concentration of probiotic to nevertheless be successful, as competition was low. It was also thought that a smaller dose would be more cost effective and more easily administered in a farm setting.

While protozoal densities increased over time until they plateaued, methanogen densities varied greatly in the first six months of life for lambs. This is likely due to the changing diet and commensal/competitive bacterial populations in the rumen. There was some correlation at weeks 1 and 23 between densities, but it likely that these factors affected one taxon independently of the other despite their general symbiosis. When methanogen density decreased at week 6, the proportion of acetogenic bacteria increased (i.e. phylum Actinobacteria, and species such as *Acetivibrio* and *Acetitomaculum* in the phylum Firmicutes, class Clostridia), fostering competitive pathways to methanogenesis [80]. There was previously shown to be a positive correlation between bacterial Fibrobacteraceae and archaeal *Methanobrevibacter ruminantium* [81]; however, in the previous study there was a negative correlation between Fibrobacteraceae and methanogen density at week 11. Reducing methanogenesis, though not investigated here, would not only make for more eco-friendly livestock, but would also reduce the amount of energy lost to the host which might have otherwise been used for production.

## Conclusions

Neither weight gain nor wool quality was improved in lambs given a probiotic, however, dietary efficiency was slightly increased as evidenced by the reduced feed intake (and rearing costs) without a significant loss to weight gain. Experimental lambs had a lower ratio of acetate to propionate than control lambs, which has previously been shown to indicate increased dietary efficiency. Despite the small increase in dietary efficiency, a more dramatic increase in production might result from altering the probiotic administered in the present study. Increasing the dosage, using a different mix of fibrolytic bacteria, or using a probiotic with fibrolytic and lactic-acid bacterial strains, are all potential methods of improving upon the results presented here.

## Supporting Information

S1 Table The genera identified and their respective abundance per sample. (PDF)
