# Supplementary material for: Fibrolytic Bacteria Isolated from the Rumen of North American Moose (Alces alces) and Their Use as a Probiotic in Neonatal Lambs
Source: PLoS One. 2015 Dec 30;10(12):e0144804. doi: 10.1371/journal.pone.0144804 (PMC4696820; doi:10.1371/journal.pone.0144804)
Supplement: S1 Table — (PDF) [file pone.0144804.s001.pdf]

taxon	Exp wk 2 sum	Exp wk 2 %	Con wk 2 sum	Con wk 2 %	Exp wk 6 sum	Exp wk 6 %	Con wk 6 sum	Con wk 6 %	Exp wk 11 sum	Exp wk 11 %	Con wk 11 sum	Con wk 11 %	Exp wk 23 sum	Exp wk 23 %	Con wk 23 sum	Con wk 23 %
A_51	0	0.0%	2	0.0%	8	0.0%	2	0.0%	9	0.0%	9	0.0%	7	0.0%	13	0.0%
Abiotrophia	0	0.0%	0	0.0%	0	0.0%	1	0.0%	0	0.0%	0	0.0%	0	0.0%	0	0.0%
Acetanaerobacterium	54	0.0%	4	0.0%	28	0.0%	51	0.0%	33	0.0%	31	0.0%	41	0.0%	78	0.0%
Acetitomaculum	11	0.0%	7	0.0%	9619	2.1%	3623	0.7%	213	0.1%	373	0.1%	523	0.1%	643	0.1%
Acetivibrio	8	0.0%	3	0.0%	893	0.2%	147	0.0%	66	0.0%	52	0.0%	282	0.1%	392	0.1%
Acetobacterium	0	0.0%	1	0.0%	0	0.0%	0	0.0%	0	0.0%	0	0.0%	1	0.0%	0	0.0%
Acholeplasma	27	0.0%	84	0.0%	2669	0.6%	1489	0.3%	253	0.1%	758	0.3%	2229	0.5%	871	0.2%
Acidaminobacter	43	0.0%	5	0.0%	12	0.0%	6	0.0%	7	0.0%	2	0.0%	5	0.0%	6	0.0%
Acidaminococcus	3	0.0%	0	0.0%	44	0.0%	24	0.0%	0	0.0%	1	0.0%	19	0.0%	24	0.0%
Acidimicrobineae	0	0.0%	0	0.0%	0	0.0%	0	0.0%	0	0.0%	2	0.0%	43	0.0%	20	0.0%
Acidiphilium	0	0.0%	0	0.0%	0	0.0%	0	0.0%	0	0.0%	0	0.0%	4	0.0%	3	0.0%
Acidithiobacillus	0	0.0%	0	0.0%	0	0.0%	0	0.0%	0	0.0%	2	0.0%	0	0.0%	2	0.0%
Acidobacterium	0	0.0%	0	0.0%	0	0.0%	0	0.0%	1	0.0%	0	0.0%	0	0.0%	1	0.0%
Acidocella	0	0.0%	1	0.0%	1	0.0%	1	0.0%	0	0.0%	0	0.0%	3	0.0%	3	0.0%
Acidothermus	1	0.0%	1	0.0%	20	0.0%	3	0.0%	4	0.0%	8	0.0%	28	0.0%	27	0.0%
Acidovorax	1	0.0%	0	0.0%	0	0.0%	0	0.0%	0	0.0%	0	0.0%	0	0.0%	0	0.0%
Acinetobacter	427	0.1%	127	0.0%	5	0.0%	1	0.0%	2	0.0%	4	0.0%	1	0.0%	1	0.0%
Actibacter	1	0.0%	18	0.0%	0	0.0%	0	0.0%	0	0.0%	1	0.0%	0	0.0%	0	0.0%
Actinoalloteichus	0	0.0%	0	0.0%	0	0.0%	0	0.0%	0	0.0%	0	0.0%	0	0.0%	1	0.0%
Actinobacillus	394	0.1%	229	0.1%	1	0.0%	1	0.0%	2	0.0%	1	0.0%	18	0.0%	17	0.0%
Actinobaculum	0	0.0%	20	0.0%	0	0.0%	0	0.0%	0	0.0%	1	0.0%	4	0.0%	4	0.0%
Actinocorallia	0	0.0%	0	0.0%	20	0.0%	11	0.0%	1	0.0%	2	0.0%	1	0.0%	5	0.0%
Actinokineospora	0	0.0%	0	0.0%	15	0.0%	0	0.0%	2	0.0%	4	0.0%	2	0.0%	1	0.0%
Actinomadura	0	0.0%	0	0.0%	0	0.0%	0	0.0%	1	0.0%	0	0.0%	1	0.0%	0	0.0%
Actinomyces	55	0.0%	7	0.0%	0	0.0%	0	0.0%	0	0.0%	2	0.0%	2	0.0%	0	0.0%
Actinosynnema	0	0.0%	0	0.0%	0	0.0%	0	0.0%	0	0.0%	0	0.0%	1	0.0%	1	0.0%
Actinotalea	0	0.0%	1	0.0%	0	0.0%	0	0.0%	0	0.0%	0	0.0%	1	0.0%	3	0.0%
Adlercreutzia	26	0.0%	14	0.0%	22	0.0%	38	0.0%	71	0.0%	64	0.0%	145	0.0%	139	0.0%
Aerococcus	2	0.0%	1	0.0%	2	0.0%	2	0.0%	0	0.0%	4	0.0%	0	0.0%	1	0.0%
Aeromicrobium	0	0.0%	0	0.0%	0	0.0%	0	0.0%	1	0.0%	0	0.0%	0	0.0%	1	0.0%
Aeromonas	0	0.0%	0	0.0%	0	0.0%	2	0.0%	0	0.0%	0	0.0%	1	0.0%	0	0.0%

Aestuariibacter	0	0.0%	0	0.0%	0	0.0%	0	0.0%	1	0.0%	0	0.0%	0	0.0%	0	0.0%
Aestuariimicrobium	0	0.0%	0	0.0%	0	0.0%	0	0.0%	1	0.0%	0	0.0%	0	0.0%	1	0.0%
Afipia	0	0.0%	0	0.0%	0	0.0%	0	0.0%	1	0.0%	0	0.0%	2	0.0%	2	0.0%
Aggregatibacter	1116	0.2%	174	0.0%	0	0.0%	0	0.0%	4	0.0%	3	0.0%	2	0.0%	2	0.0%
Agreia	0	0.0%	0	0.0%	0	0.0%	0	0.0%	1	0.0%	0	0.0%	2	0.0%	0	0.0%
Agrococcus	0	0.0%	0	0.0%	0	0.0%	0	0.0%	0	0.0%	0	0.0%	0	0.0%	3	0.0%
Agromyces	0	0.0%	0	0.0%	0	0.0%	0	0.0%	0	0.0%	0	0.0%	6	0.0%	5	0.0%
Akkermansia	5	0.0%	1026	0.2%	0	0.0%	0	0.0%	6	0.0%	2	0.0%	0	0.0%	0	0.0%
Alcanivorax	0	0.0%	0	0.0%	0	0.0%	0	0.0%	0	0.0%	0	0.0%	0	0.0%	1	0.0%
Algoriphagus	0	0.0%	0	0.0%	0	0.0%	1	0.0%	0	0.0%	0	0.0%	0	0.0%	2	0.0%
Alicycliphilus	0	0.0%	0	0.0%	0	0.0%	0	0.0%	0	0.0%	0	0.0%	0	0.0%	1	0.0%
Alicyclobacillus	0	0.0%	2	0.0%	1105	0.2%	39	0.0%	5	0.0%	5	0.0%	2	0.0%	4	0.0%
Alistipes	20	0.0%	4	0.0%	3	0.0%	1	0.0%	47	0.0%	3	0.0%	17	0.0%	21	0.0%
Alkalibacterium	0	0.0%	0	0.0%	0	0.0%	0	0.0%	0	0.0%	0	0.0%	2	0.0%	3	0.0%
Alkalilimnicola	0	0.0%	0	0.0%	0	0.0%	0	0.0%	0	0.0%	1	0.0%	0	0.0%	0	0.0%
Alkalimonas	0	0.0%	1	0.0%	0	0.0%	0	0.0%	0	0.0%	0	0.0%	1	0.0%	0	0.0%
Allobaculum	0	0.0%	0	0.0%	0	0.0%	0	0.0%	0	0.0%	0	0.0%	0	0.0%	1	0.0%
Allochromatium	0	0.0%	0	0.0%	0	0.0%	1	0.0%	0	0.0%	1	0.0%	0	0.0%	1	0.0%
Alloiococcus	0	0.0%	0	0.0%	0	0.0%	0	0.0%	0	0.0%	0	0.0%	4	0.0%	0	0.0%
Alloscardovia	0	0.0%	0	0.0%	0	0.0%	0	0.0%	7	0.0%	10	0.0%	3	0.0%	8	0.0%
Alteromonas	0	0.0%	0	0.0%	2	0.0%	0	0.0%	0	0.0%	0	0.0%	2	0.0%	2	0.0%
Alysiella	378	0.1%	128	0.0%	0	0.0%	1	0.0%	3	0.0%	3	0.0%	0	0.0%	1	0.0%
Aminiphilus	11	0.0%	15	0.0%	0	0.0%	0	0.0%	0	0.0%	0	0.0%	0	0.0%	0	0.0%
Aminobacter	0	0.0%	0	0.0%	0	0.0%	0	0.0%	0	0.0%	1	0.0%	3	0.0%	1	0.0%
Aminobacterium	0	0.0%	0	0.0%	0	0.0%	0	0.0%	0	0.0%	0	0.0%	1	0.0%	0	0.0%
Ammoniphilus	0	0.0%	3	0.0%	0	0.0%	2	0.0%	1	0.0%	1	0.0%	1	0.0%	1	0.0%
Anaeroarcus- Anaeromusa	0	0.0%	0	0.0%	0	0.0%	0	0.0%	1	0.0%	0	0.0%	45	0.0%	37	0.0%
Anaerobacter	0	0.0%	0	0.0%	0	0.0%	0	0.0%	9	0.0%	5	0.0%	10	0.0%	12	0.0%
Anaerobacter	7	0.0%	0	0.0%	15	0.0%	7	0.0%	1	0.0%	1	0.0%	4	0.0%	6	0.0%
Anaerobiospirillum	1	0.0%	0	0.0%	14	0.0%	59	0.0%	2	0.0%	7	0.0%	127	0.0%	142	0.0%
Anaerobranca	0	0.0%	0	0.0%	0	0.0%	0	0.0%	0	0.0%	0	0.0%	1	0.0%	0	0.0%
Anaerolinea	1	0.0%	4	0.0%	0	0.0%	0	0.0%	0	0.0%	0	0.0%	1	0.0%	3	0.0%
Anaeromyxobacter	1	0.0%	0	0.0%	0	0.0%	0	0.0%	0	0.0%	0	0.0%	0	0.0%	1	0.0%

Anaerophaga	3	0.0%	5	0.0%	6	0.0%	4	0.0%	264	0.1%	131	0.1%	292	0.1%	227	0.0%
Anaeroplasma	16	0.0%	21	0.0%	646	0.1%	1350	0.2%	3034	1.2%	848	0.3%	115	0.0%	54	0.0%
Anaerosinus	0	0.0%	0	0.0%	0	0.0%	0	0.0%	0	0.0%	0	0.0%	1	0.0%	0	0.0%
Anaerospora	0	0.0%	0	0.0%	0	0.0%	0	0.0%	2	0.0%	2	0.0%	11	0.0%	5	0.0%
Anaerostipes	62	0.0%	82	0.0%	207	0.0%	287	0.1%	372	0.1%	301	0.1%	296	0.1%	421	0.1%
Anaerovirgula	0	0.0%	0	0.0%	0	0.0%	0	0.0%	0	0.0%	0	0.0%	2	0.0%	3	0.0%
Anaerovorax	435	0.1%	489	0.1%	26	0.0%	21	0.0%	85	0.0%	49	0.0%	218	0.0%	247	0.0%
Ancylobacter	0	0.0%	0	0.0%	0	0.0%	0	0.0%	0	0.0%	0	0.0%	2	0.0%	0	0.0%
Aneurinibacillus	0	0.0%	0	0.0%	0	0.0%	0	0.0%	0	0.0%	0	0.0%	1	0.0%	0	0.0%
Angulomicrobium	0	0.0%	0	0.0%	0	0.0%	0	0.0%	0	0.0%	0	0.0%	1	0.0%	0	0.0%
Anoxybacillus	0	0.0%	0	0.0%	0	0.0%	0	0.0%	0	0.0%	0	0.0%	2	0.0%	3	0.0%
Anoxybacillus	1	0.0%	0	0.0%	0	0.0%	1	0.0%	0	0.0%	1	0.0%	0	0.0%	0	0.0%
Aquamonas	0	0.0%	0	0.0%	0	0.0%	0	0.0%	0	0.0%	0	0.0%	3	0.0%	0	0.0%
Aquicella	0	0.0%	0	0.0%	0	0.0%	0	0.0%	0	0.0%	0	0.0%	76	0.0%	63	0.0%
Aquimarina	0	0.0%	0	0.0%	0	0.0%	0	0.0%	1	0.0%	2	0.0%	0	0.0%	0	0.0%
Arcanobacterium	0	0.0%	14	0.0%	0	0.0%	0	0.0%	0	0.0%	0	0.0%	1	0.0%	2	0.0%
Arcobacter	0	0.0%	1	0.0%	0	0.0%	0	0.0%	0	0.0%	0	0.0%	1	0.0%	1	0.0%
Arenibacter	0	0.0%	2	0.0%	0	0.0%	0	0.0%	0	0.0%	0	0.0%	1	0.0%	4	0.0%
Arenimonas	0	0.0%	0	0.0%	0	0.0%	0	0.0%	0	0.0%	0	0.0%	1	0.0%	2	0.0%
Arhtrobacter	0	0.0%	1	0.0%	8	0.0%	0	0.0%	0	0.0%	1	0.0%	0	0.0%	2	0.0%
Aspromonas	0	0.0%	1	0.0%	0	0.0%	0	0.0%	0	0.0%	0	0.0%	0	0.0%	0	0.0%
Asticcacaulis	0	0.0%	0	0.0%	0	0.0%	0	0.0%	0	0.0%	0	0.0%	0	0.0%	1	0.0%
Atopobacter	7	0.0%	2	0.0%	1	0.0%	0	0.0%	0	0.0%	0	0.0%	0	0.0%	0	0.0%
Atopobium	78	0.0%	129	0.0%	74	0.0%	219	0.0%	25	0.0%	49	0.0%	92	0.0%	113	0.0%
Avibacterium	9	0.0%	0	0.0%	0	0.0%	0	0.0%	0	0.0%	0	0.0%	0	0.0%	1	0.0%
Azoarcus	41	0.0%	6	0.0%	0	0.0%	0	0.0%	1	0.0%	1	0.0%	1	0.0%	0	0.0%
Azonexus	75	0.0%	11	0.0%	0	0.0%	0	0.0%	2	0.0%	1	0.0%	3	0.0%	3	0.0%
Azorhizobium	0	0.0%	0	0.0%	1	0.0%	0	0.0%	0	0.0%	0	0.0%	0	0.0%	0	0.0%
Azospirillum	0	0.0%	0	0.0%	0	0.0%	0	0.0%	0	0.0%	0	0.0%	20	0.0%	18	0.0%
Azotobacter	0	0.0%	0	0.0%	0	0.0%	0	0.0%	0	0.0%	0	0.0%	1	0.0%	0	0.0%
Bacillus	23	0.0%	15	0.0%	565	0.1%	28	0.0%	7	0.0%	16	0.0%	12	0.0%	25	0.0%
Bacteriovorax	0	0.0%	0	0.0%	0	0.0%	0	0.0%	0	0.0%	0	0.0%	2	0.0%	2	0.0%
Bacteroides	19644	3.7%	57907	13.6%	282	0.1%	594	0.1%	3038	1.2%	2555	1.0%	6071	1.3%	8234	1.6%
Balneatrix	0	0.0%	0	0.0%	1	0.0%	9	0.0%	2	0.0%	5	0.0%	16	0.0%	13	0.0%

Barnesiella	9	0.0%	7	0.0%	76	0.0%	73	0.0%	674	0.3%	191	0.1%	407	0.1%	511	0.1%
Bartonella	0	0.0%	1	0.0%	0	0.0%	0	0.0%	0	0.0%	0	0.0%	11	0.0%	15	0.0%
BD1-7	0	0.0%	0	0.0%	0	0.0%	0	0.0%	1	0.0%	0	0.0%	1	0.0%	0	0.0%
Bdellovibrio	0	0.0%	0	0.0%	0	0.0%	0	0.0%	1	0.0%	0	0.0%	17	0.0%	11	0.0%
Beijerinckia	2	0.0%	0	0.0%	0	0.0%	0	0.0%	0	0.0%	0	0.0%	0	0.0%	0	0.0%
Belliella	3	0.0%	0	0.0%	0	0.0%	0	0.0%	0	0.0%	0	0.0%	0	0.0%	1	0.0%
Bellilinea	0	0.0%	0	0.0%	0	0.0%	0	0.0%	0	0.0%	1	0.0%	0	0.0%	1	0.0%
Bergeriella	98	0.0%	4	0.0%	0	0.0%	1	0.0%	8	0.0%	5	0.0%	1	0.0%	1	0.0%
Bergeyella	5	0.0%	0	0.0%	0	0.0%	0	0.0%	0	0.0%	0	0.0%	0	0.0%	0	0.0%
Bifidobacterium	2	0.0%	2	0.0%	99	0.0%	898	0.2%	4	0.0%	8	0.0%	5	0.0%	13	0.0%
Bilophila	0	0.0%	2	0.0%	0	0.0%	1	0.0%	3	0.0%	8	0.0%	32	0.0%	16	0.0%
Blastobacter	0	0.0%	1	0.0%	0	0.0%	0	0.0%	0	0.0%	0	0.0%	0	0.0%	1	0.0%
Blastococcus	0	0.0%	0	0.0%	0	0.0%	0	0.0%	0	0.0%	0	0.0%	1	0.0%	0	0.0%
Blastopirellula	13	0.0%	6	0.0%	2	0.0%	9	0.0%	7	0.0%	12	0.0%	39	0.0%	42	0.0%
Blautia	708	0.1%	9876	2.3%	4912	1.1%	10455	1.9%	664	0.3%	648	0.3%	1233	0.3%	2135	0.4%
Blvii28	7	0.0%	4	0.0%	6	0.0%	3	0.0%	49	0.0%	54	0.0%	266	0.1%	241	0.0%
Bosea	0	0.0%	0	0.0%	0	0.0%	0	0.0%	0	0.0%	1	0.0%	7	0.0%	5	0.0%
Bowmanella	0	0.0%	0	0.0%	0	0.0%	0	0.0%	0	0.0%	0	0.0%	1	0.0%	2	0.0%
Brachybacterium	0	0.0%	0	0.0%	0	0.0%	0	0.0%	0	0.0%	0	0.0%	2	0.0%	1	0.0%
Brachymonas	2	0.0%	0	0.0%	0	0.0%	0	0.0%	0	0.0%	1	0.0%	1	0.0%	1	0.0%
Bradyrhizobium	0	0.0%	1	0.0%	0	0.0%	2	0.0%	3	0.0%	1	0.0%	4	0.0%	3	0.0%
Brenneria	0	0.0%	0	0.0%	0	0.0%	4	0.0%	0	0.0%	0	0.0%	1	0.0%	0	0.0%
Brevibacillus	7	0.0%	4	0.0%	85	0.0%	15	0.0%	11	0.0%	21	0.0%	3	0.0%	6	0.0%
Brevibacterium	2	0.0%	0	0.0%	0	0.0%	1	0.0%	2	0.0%	3	0.0%	7	0.0%	8	0.0%
Brevundimonas	1	0.0%	0	0.0%	0	0.0%	0	0.0%	2	0.0%	5	0.0%	2	0.0%	2	0.0%
Brochothrix	0	0.0%	2	0.0%	2	0.0%	0	0.0%	0	0.0%	0	0.0%	0	0.0%	0	0.0%
Brumimimicrobium	0	0.0%	3	0.0%	3	0.0%	1	0.0%	3	0.0%	5	0.0%	3	0.0%	5	0.0%
Budvicia	1	0.0%	0	0.0%	3	0.0%	1	0.0%	0	0.0%	0	0.0%	0	0.0%	0	0.0%
Bulleidia	5	0.0%	7	0.0%	1	0.0%	6	0.0%	10	0.0%	4	0.0%	8	0.0%	16	0.0%
Burkholderia	0	0.0%	0	0.0%	0	0.0%	0	0.0%	1	0.0%	2	0.0%	5	0.0%	10	0.0%
Butyrivibrio	912	0.2%	443	0.1%	13907	3.1%	2583	0.5%	7054	2.8%	3759	1.5%	5620	1.2%	13564	2.6%
Butyrivibrio-Pseudobutyrivibrio	42	0.0%	86	0.0%	846	0.2%	734	0.1%	1334	0.5%	971	0.4%	2584	0.5%	5411	1.0%
Caenispirillum	0	0.0%	0	0.0%	0	0.0%	0	0.0%	0	0.0%	0	0.0%	1	0.0%	1	0.0%

Caldalkalibacillus	0	0.0%	0	0.0%	0	0.0%	0	0.0%	0	0.0%	0	0.0%	0	0.0%	2	0.0%
Caldanaerobacter	0	0.0%	0	0.0%	0	0.0%	0	0.0%	0	0.0%	0	0.0%	2	0.0%	3	0.0%
Caldanaerobius	140	0.0%	42	0.0%	0	0.0%	0	0.0%	3	0.0%	2	0.0%	5	0.0%	3	0.0%
Caldilinea	0	0.0%	1	0.0%	0	0.0%	0	0.0%	0	0.0%	1	0.0%	6	0.0%	5	0.0%
Caldimonas	1	0.0%	0	0.0%	0	0.0%	0	0.0%	0	0.0%	0	0.0%	0	0.0%	1	0.0%
Calditerrivibrio	0	0.0%	0	0.0%	0	0.0%	0	0.0%	0	0.0%	0	0.0%	0	0.0%	1	0.0%
Caloramator	0	0.0%	0	0.0%	2	0.0%	0	0.0%	3	0.0%	2	0.0%	7	0.0%	5	0.0%
Caminibacter	2	0.0%	0	0.0%	0	0.0%	0	0.0%	1	0.0%	0	0.0%	10	0.0%	6	0.0%
Caminicella	7	0.0%	0	0.0%	1	0.0%	0	0.0%	5	0.0%	4	0.0%	4	0.0%	6	0.0%
Campylobacter	17	0.0%	8	0.0%	4	0.0%	0	0.0%	15	0.0%	1	0.0%	4	0.0%	3	0.0%
Cand. Amoebinatus	0	0.0%	4	0.0%	0	0.0%	0	0.0%	0	0.0%	0	0.0%	0	0.0%	0	0.0%
Cand. Aquiluna	0	0.0%	1	0.0%	0	0.0%	0	0.0%	0	0.0%	2	0.0%	3	0.0%	3	0.0%
Cand. Arthromitus	0	0.0%	0	0.0%	2	0.0%	1	0.0%	1	0.0%	3	0.0%	5610	1.2%	3121	0.6%
Cand. Contubernalis	0	0.0%	0	0.0%	0	0.0%	0	0.0%	0	0.0%	1	0.0%	3	0.0%	0	0.0%
Cand. Limnoluna	0	0.0%	7	0.0%	0	0.0%	0	0.0%	0	0.0%	2	0.0%	13	0.0%	15	0.0%
Cand. Magnetobacterium	0	0.0%	0	0.0%	0	0.0%	0	0.0%	0	0.0%	0	0.0%	6	0.0%	3	0.0%
Cand. Planktoluna	0	0.0%	1	0.0%	1	0.0%	1	0.0%	0	0.0%	2	0.0%	10	0.0%	10	0.0%
Cand. Protochlamydia	0	0.0%	0	0.0%	0	0.0%	0	0.0%	0	0.0%	0	0.0%	1	0.0%	0	0.0%
Cand. Rhodoluna	1	0.0%	3	0.0%	0	0.0%	0	0.0%	0	0.0%	0	0.0%	22	0.0%	10	0.0%
Cand. Symbiothrix	0	0.0%	0	0.0%	0	0.0%	0	0.0%	0	0.0%	2	0.0%	0	0.0%	6	0.0%
Cand. Tammella	3	0.0%	1	0.0%	0	0.0%	0	0.0%	7	0.0%	16	0.0%	184	0.0%	205	0.0%
Capnocytophaga	58	0.0%	71	0.0%	0	0.0%	0	0.0%	12	0.0%	7	0.0%	5	0.0%	1	0.0%
Carnobacterium	0	0.0%	0	0.0%	0	0.0%	0	0.0%	2	0.0%	1	0.0%	6	0.0%	4	0.0%
Catabacter	352	0.1%	12317	2.9%	942	0.2%	141	0.0%	230	0.1%	229	0.1%	334	0.1%	357	0.1%
Catenibacterium	1	0.0%	8	0.0%	9961	2.2%	408	0.1%	51	0.0%	26	0.0%	36	0.0%	41	0.0%
Catenulispora	0	0.0%	0	0.0%	0	0.0%	0	0.0%	0	0.0%	0	0.0%	1	0.0%	0	0.0%
Catenulisporineae	0	0.0%	0	0.0%	0	0.0%	0	0.0%	0	0.0%	0	0.0%	1	0.0%	0	0.0%
Catonella	1	0.0%	2	0.0%	7	0.0%	5	0.0%	16	0.0%	15	0.0%	117	0.0%	142	0.0%
Caulobacter	0	0.0%	0	0.0%	1	0.0%	0	0.0%	0	0.0%	0	0.0%	1	0.0%	0	0.0%
Cellulomonas	1	0.0%	0	0.0%	1	0.0%	0	0.0%	1	0.0%	3	0.0%	17	0.0%	36	0.0%
Cellulophaga	0	0.0%	1	0.0%	0	0.0%	0	0.0%	0	0.0%	0	0.0%	0	0.0%	0	0.0%
Cellulosimicrobium	0	0.0%	0	0.0%	0	0.0%	1	0.0%	0	0.0%	0	0.0%	2	0.0%	3	0.0%
Cellvibrio	0	0.0%	0	0.0%	0	0.0%	0	0.0%	0	0.0%	0	0.0%	105	0.0%	134	0.0%
Cerasibacillus	5	0.0%	1	0.0%	1	0.0%	0	0.0%	5	0.0%	6	0.0%	0	0.0%	2	0.0%

Cetobacterium	1	0.0%	2	0.0%	0	0.0%	0	0.0%	0	0.0%	0	0.0%	0	0.0%	0	0.0%
Chelatococcus	0	0.0%	0	0.0%	0	0.0%	0	0.0%	0	0.0%	0	0.0%	0	0.0%	1	0.0%
Chitinimonas	1	0.0%	0	0.0%	0	0.0%	0	0.0%	0	0.0%	0	0.0%	0	0.0%	0	0.0%
Chlorobium	0	0.0%	0	0.0%	0	0.0%	0	0.0%	0	0.0%	0	0.0%	1	0.0%	0	0.0%
Chryseobacterium	4	0.0%	2	0.0%	1	0.0%	0	0.0%	1	0.0%	2	0.0%	0	0.0%	0	0.0%
Clavibacter	0	0.0%	0	0.0%	0	0.0%	0	0.0%	0	0.0%	0	0.0%	0	0.0%	1	0.0%
Clostridium	63	0.0%	85	0.0%	194	0.0%	56	0.0%	11	0.0%	3	0.0%	5769	1.2%	3336	0.6%
Cobetia	0	0.0%	1	0.0%	0	0.0%	0	0.0%	0	0.0%	0	0.0%	0	0.0%	0	0.0%
Cohnella	0	0.0%	0	0.0%	0	0.0%	0	0.0%	0	0.0%	0	0.0%	2	0.0%	0	0.0%
Collimonas	0	0.0%	0	0.0%	0	0.0%	0	0.0%	1	0.0%	0	0.0%	0	0.0%	0	0.0%
Collinsella	0	0.0%	0	0.0%	0	0.0%	37	0.0%	1	0.0%	2	0.0%	0	0.0%	0	0.0%
Colwellia	0	0.0%	0	0.0%	0	0.0%	0	0.0%	0	0.0%	1	0.0%	0	0.0%	0	0.0%
Comamonas	1	0.0%	1	0.0%	0	0.0%	0	0.0%	0	0.0%	0	0.0%	1	0.0%	0	0.0%
Conchiformibius	66	0.0%	19	0.0%	1	0.0%	0	0.0%	0	0.0%	0	0.0%	0	0.0%	0	0.0%
Coprococcus	360	0.1%	195	0.0%	369	0.1%	418	0.1%	829	0.3%	697	0.3%	2810	0.6%	5134	1.0%
Coriobacterium	0	0.0%	0	0.0%	0	0.0%	1	0.0%	0	0.0%	0	0.0%	0	0.0%	0	0.0%
Corynebacterium	2	0.0%	7	0.0%	1	0.0%	0	0.0%	339	0.1%	376	0.2%	26	0.0%	19	0.0%
Croceibacter	0	0.0%	0	0.0%	0	0.0%	0	0.0%	0	0.0%	0	0.0%	0	0.0%	1	0.0%
Crocinitomix	0	0.0%	2	0.0%	0	0.0%	0	0.0%	0	0.0%	0	0.0%	1	0.0%	0	0.0%
Cryptanaerobacter	2	0.0%	1	0.0%	0	0.0%	0	0.0%	27	0.0%	1	0.0%	9	0.0%	11	0.0%
Cryptosporangium	0	0.0%	0	0.0%	0	0.0%	0	0.0%	0	0.0%	1	0.0%	0	0.0%	0	0.0%
Cupriavidus	0	0.0%	1	0.0%	0	0.0%	0	0.0%	0	0.0%	0	0.0%	3	0.0%	6	0.0%
Curtobacterium	0	0.0%	0	0.0%	0	0.0%	1	0.0%	2	0.0%	0	0.0%	4	0.0%	1	0.0%
Cyclobacterium	1	0.0%	0	0.0%	0	0.0%	0	0.0%	0	0.0%	0	0.0%	7	0.0%	16	0.0%
Cycloclasticus	0	0.0%	0	0.0%	0	0.0%	0	0.0%	1	0.0%	1	0.0%	0	0.0%	0	0.0%
Cytophaga	2	0.0%	0	0.0%	0	0.0%	0	0.0%	0	0.0%	2	0.0%	0	0.0%	1	0.0%
Dechloromonas	2	0.0%	0	0.0%	0	0.0%	0	0.0%	0	0.0%	0	0.0%	1	0.0%	3	0.0%
Deferribacter	0	0.0%	0	0.0%	0	0.0%	0	0.0%	0	0.0%	0	0.0%	0	0.0%	1	0.0%
Defluviicoccus	0	0.0%	0	0.0%	0	0.0%	0	0.0%	0	0.0%	6	0.0%	3	0.0%	6	0.0%
Dehalobacter	2	0.0%	1	0.0%	0	0.0%	0	0.0%	1	0.0%	0	0.0%	1	0.0%	2	0.0%
Demequina	0	0.0%	0	0.0%	0	0.0%	0	0.0%	1	0.0%	0	0.0%	4	0.0%	1	0.0%
Dermatophilus	0	0.0%	0	0.0%	0	0.0%	0	0.0%	0	0.0%	0	0.0%	4	0.0%	3	0.0%
Derxia	6	0.0%	1	0.0%	0	0.0%	1	0.0%	0	0.0%	0	0.0%	5	0.0%	11	0.0%
Desulfatiferula	0	0.0%	0	0.0%	0	0.0%	0	0.0%	0	0.0%	1	0.0%	0	0.0%	0	0.0%

Desulfitobacterium	1	0.0%	0	0.0%	1	0.0%	0	0.0%	0	0.0%	0	0.0%	0	0.0%	2	0.0%
Desulfobacca	0	0.0%	0	0.0%	0	0.0%	0	0.0%	0	0.0%	1	0.0%	0	0.0%	0	0.0%
Desulfobacterium	0	0.0%	0	0.0%	0	0.0%	0	0.0%	1	0.0%	0	0.0%	1	0.0%	0	0.0%
Desulfobulbus	0	0.0%	0	0.0%	0	0.0%	0	0.0%	0	0.0%	0	0.0%	10	0.0%	6	0.0%
Desulfomicrobium	0	0.0%	0	0.0%	0	0.0%	0	0.0%	0	0.0%	0	0.0%	1	0.0%	0	0.0%
Desulfomonile	0	0.0%	0	0.0%	0	0.0%	0	0.0%	1	0.0%	0	0.0%	1	0.0%	1	0.0%
Desulfonatronum	0	0.0%	0	0.0%	0	0.0%	0	0.0%	0	0.0%	0	0.0%	1	0.0%	0	0.0%
Desulfonauticus	0	0.0%	0	0.0%	0	0.0%	0	0.0%	0	0.0%	1	0.0%	5	0.0%	7	0.0%
Desulfonema	0	0.0%	0	0.0%	0	0.0%	0	0.0%	0	0.0%	0	0.0%	0	0.0%	1	0.0%
Desulfosporosinus	3	0.0%	1	0.0%	1	0.0%	0	0.0%	6	0.0%	1	0.0%	4	0.0%	7	0.0%
Desulfotalea	0	0.0%	0	0.0%	0	0.0%	0	0.0%	0	0.0%	0	0.0%	0	0.0%	1	0.0%
Desulfotomaculum	12	0.0%	65	0.0%	1	0.0%	0	0.0%	18	0.0%	1	0.0%	14	0.0%	11	0.0%
Desulfovibrio	139	0.0%	83	0.0%	83	0.0%	69	0.0%	125	0.0%	99	0.0%	1017	0.2%	910	0.2%
Desulfurispora	1	0.0%	1	0.0%	0	0.0%	0	0.0%	0	0.0%	0	0.0%	0	0.0%	0	0.0%
Desulfurivibrio	0	0.0%	0	0.0%	0	0.0%	0	0.0%	0	0.0%	0	0.0%	1	0.0%	0	0.0%
Desulfurobacterium	0	0.0%	0	0.0%	0	0.0%	0	0.0%	0	0.0%	0	0.0%	0	0.0%	1	0.0%
Desulfuromonas	1	0.0%	0	0.0%	0	0.0%	0	0.0%	1	0.0%	0	0.0%	6	0.0%	4	0.0%
Desulfuromusa	0	0.0%	0	0.0%	0	0.0%	1	0.0%	0	0.0%	0	0.0%	0	0.0%	1	0.0%
Dethiobacter	1	0.0%	0	0.0%	0	0.0%	0	0.0%	3	0.0%	2	0.0%	0	0.0%	1	0.0%
Dethiosulfovibrio	10	0.0%	19	0.0%	0	0.0%	0	0.0%	1	0.0%	3	0.0%	8	0.0%	5	0.0%
Devosia	0	0.0%	0	0.0%	0	0.0%	0	0.0%	0	0.0%	4	0.0%	11	0.0%	11	0.0%
Dialister	20	0.0%	3	0.0%	0	0.0%	0	0.0%	1	0.0%	16	0.0%	12880	2.7%	13534	2.6%
Diaphorobacter	0	0.0%	1	0.0%	0	0.0%	0	0.0%	0	0.0%	0	0.0%	1	0.0%	0	0.0%
Dickeya	0	0.0%	0	0.0%	0	0.0%	2	0.0%	0	0.0%	0	0.0%	0	0.0%	0	0.0%
Dietzia	0	0.0%	1	0.0%	0	0.0%	1	0.0%	0	0.0%	1	0.0%	8	0.0%	12	0.0%
Dokdonella	0	0.0%	0	0.0%	0	0.0%	0	0.0%	0	0.0%	0	0.0%	1	0.0%	2	0.0%
Dolosicoccus	1	0.0%	0	0.0%	0	0.0%	0	0.0%	0	0.0%	0	0.0%	0	0.0%	0	0.0%
Dorea	523	0.1%	939	0.2%	69	0.0%	158	0.0%	27	0.0%	105	0.0%	157	0.0%	249	0.0%
Duganella	0	0.0%	0	0.0%	0	0.0%	0	0.0%	1	0.0%	0	0.0%	2	0.0%	0	0.0%
Dyadobacter	0	0.0%	0	0.0%	0	0.0%	0	0.0%	0	0.0%	0	0.0%	1	0.0%	2	0.0%
Dyella	0	0.0%	3	0.0%	0	0.0%	0	0.0%	1	0.0%	1	0.0%	0	0.0%	2	0.0%
Dysgonomonas	25	0.0%	11	0.0%	2	0.0%	3	0.0%	46	0.0%	22	0.0%	48	0.0%	46	0.0%
Edaphobacter	0	0.0%	0	0.0%	0	0.0%	0	0.0%	0	0.0%	0	0.0%	1	0.0%	0	0.0%
Eggerthella	10	0.0%	11	0.0%	59	0.0%	82	0.0%	32	0.0%	65	0.0%	93	0.0%	142	0.0%

Ehrlichia	0	0.0%	0	0.0%	0	0.0%	0	0.0%	0	0.0%	0	0.0%	0	0.0%	1	0.0%
Elizabethkingia	1	0.0%	3	0.0%	0	0.0%	0	0.0%	0	0.0%	0	0.0%	0	0.0%	0	0.0%
Empedobacter	1	0.0%	0	0.0%	0	0.0%	0	0.0%	0	0.0%	0	0.0%	0	0.0%	0	0.0%
Ensifer	0	0.0%	0	0.0%	0	0.0%	0	0.0%	0	0.0%	0	0.0%	1	0.0%	0	0.0%
Enteric_Bacteria_cluster	32	0.0%	17	0.0%	19	0.0%	7	0.0%	0	0.0%	1	0.0%	11	0.0%	22	0.0%
Enterococcus	17	0.0%	34	0.0%	2	0.0%	4	0.0%	5	0.0%	13	0.0%	1	0.0%	11	0.0%
Enterovibrio	9	0.0%	0	0.0%	0	0.0%	1	0.0%	0	0.0%	0	0.0%	0	0.0%	0	0.0%
Entomoplasma	0	0.0%	0	0.0%	1	0.0%	0	0.0%	2	0.0%	3	0.0%	3	0.0%	3	0.0%
Epulopiscium	16	0.0%	2	0.0%	8	0.0%	1	0.0%	14	0.0%	3	0.0%	24	0.0%	29	0.0%
Eremococcus	1	0.0%	1	0.0%	0	0.0%	0	0.0%	0	0.0%	0	0.0%	0	0.0%	0	0.0%
Erwinia	0	0.0%	1	0.0%	0	0.0%	0	0.0%	0	0.0%	0	0.0%	0	0.0%	0	0.0%
Erysipelothrix	258	0.0%	1255	0.3%	11	0.0%	34	0.0%	35	0.0%	83	0.0%	28	0.0%	36	0.0%
Erythrobacter	0	0.0%	0	0.0%	0	0.0%	0	0.0%	1	0.0%	0	0.0%	7	0.0%	10	0.0%
Escherichia	10	0.0%	6	0.0%	5	0.0%	0	0.0%	2	0.0%	2	0.0%	2	0.0%	3	0.0%
Ethanoligenens	9	0.0%	13	0.0%	5	0.0%	3	0.0%	22	0.0%	8	0.0%	13	0.0%	28	0.0%
Eubacterium	2721	0.5%	922	0.2%	137	0.0%	115	0.0%	282	0.1%	154	0.1%	280	0.1%	378	0.1%
Exiguobacterium	0	0.0%	0	0.0%	2	0.0%	0	0.0%	1	0.0%	3	0.0%	1	0.0%	1	0.0%
Faecalibacterium	69	0.0%	31	0.0%	405	0.1%	174	0.0%	546	0.2%	2503	1.0%	633	0.1%	935	0.2%
Fastidiosipila	1281	0.2%	1332	0.3%	1332	0.3%	118	0.0%	285	0.1%	510	0.2%	913	0.2%	1247	0.2%
Fervidobacterium	3	0.0%	93	0.0%	0	0.0%	0	0.0%	1	0.0%	1	0.0%	0	0.0%	0	0.0%
Filifactor	1122	0.2%	175	0.0%	0	0.0%	0	0.0%	5	0.0%	4	0.0%	3	0.0%	2	0.0%
Finegoldia	4	0.0%	12	0.0%	1	0.0%	2	0.0%	3	0.0%	13	0.0%	3584	0.7%	1850	0.4%
Flacklamia	1	0.0%	0	0.0%	0	0.0%	0	0.0%	0	0.0%	0	0.0%	0	0.0%	0	0.0%
Flammeovirga	1	0.0%	0	0.0%	0	0.0%	0	0.0%	0	0.0%	0	0.0%	0	0.0%	0	0.0%
Flavobacterium	97	0.0%	75	0.0%	1	0.0%	1	0.0%	8	0.0%	6	0.0%	15	0.0%	23	0.0%
Flexibacter	1	0.0%	0	0.0%	0	0.0%	0	0.0%	0	0.0%	0	0.0%	3	0.0%	7	0.0%
Flexithrix	9	0.0%	12	0.0%	0	0.0%	0	0.0%	4	0.0%	2	0.0%	6	0.0%	8	0.0%
Fluviicola	27	0.0%	20	0.0%	0	0.0%	1	0.0%	0	0.0%	1	0.0%	7	0.0%	16	0.0%
Fodinicola	0	0.0%	0	0.0%	0	0.0%	0	0.0%	0	0.0%	0	0.0%	0	0.0%	2	0.0%
Francisella	1	0.0%	1	0.0%	2	0.0%	0	0.0%	0	0.0%	0	0.0%	1	0.0%	3	0.0%
Frankia	0	0.0%	0	0.0%	0	0.0%	0	0.0%	1	0.0%	0	0.0%	2	0.0%	1	0.0%
Friedmanniella	0	0.0%	1	0.0%	0	0.0%	0	0.0%	0	0.0%	0	0.0%	8	0.0%	4	0.0%
Frigoribacterium	1	0.0%	0	0.0%	0	0.0%	0	0.0%	7	0.0%	11	0.0%	3	0.0%	1	0.0%
Fructobacillus	2	0.0%	1	0.0%	0	0.0%	0	0.0%	0	0.0%	0	0.0%	0	0.0%	0	0.0%

Fulvimonas	0	0.0%	0	0.0%	0	0.0%	0	0.0%	0	0.0%	0	0.0%	0	0.0%	1	0.0%
Fulvivirga	1	0.0%	0	0.0%	0	0.0%	0	0.0%	0	0.0%	0	0.0%	2	0.0%	1	0.0%
Fusibacter	118	0.0%	19	0.0%	18	0.0%	0	0.0%	2	0.0%	2	0.0%	20	0.0%	23	0.0%
Fusobacterium	353	0.1%	1012	0.2%	0	0.0%	0	0.0%	6	0.0%	10	0.0%	0	0.0%	0	0.0%
Galbibacter	0	0.0%	0	0.0%	0	0.0%	0	0.0%	7	0.0%	0	0.0%	2	0.0%	2	0.0%
Gardnerella	0	0.0%	0	0.0%	0	0.0%	0	0.0%	0	0.0%	0	0.0%	3	0.0%	4	0.0%
Gelidibacter	0	0.0%	0	0.0%	0	0.0%	0	0.0%	0	0.0%	0	0.0%	2	0.0%	6	0.0%
Gelria	3	0.0%	3	0.0%	0	0.0%	0	0.0%	2	0.0%	0	0.0%	3	0.0%	1	0.0%
Gemella	0	0.0%	0	0.0%	12	0.0%	1	0.0%	0	0.0%	1	0.0%	5	0.0%	11	0.0%
Geoalkalibacter	0	0.0%	0	0.0%	0	0.0%	0	0.0%	5	0.0%	8	0.0%	15	0.0%	20	0.0%
Geobacillus	2	0.0%	1	0.0%	1	0.0%	3	0.0%	1	0.0%	5	0.0%	6	0.0%	3	0.0%
Geobacter	4	0.0%	1	0.0%	0	0.0%	22	0.0%	28	0.0%	20	0.0%	82	0.0%	58	0.0%
Geodermatophilus	0	0.0%	0	0.0%	0	0.0%	0	0.0%	0	0.0%	0	0.0%	0	0.0%	1	0.0%
Georgenia	0	0.0%	0	0.0%	0	0.0%	0	0.0%	0	0.0%	0	0.0%	0	0.0%	1	0.0%
Geosporobacter	123	0.0%	10	0.0%	5	0.0%	6	0.0%	15	0.0%	10	0.0%	8	0.0%	11	0.0%
Gillisia	0	0.0%	0	0.0%	0	0.0%	0	0.0%	0	0.0%	0	0.0%	1	0.0%	0	0.0%
Glaciecola	0	0.0%	1	0.0%	0	0.0%	0	0.0%	0	0.0%	0	0.0%	6	0.0%	2	0.0%
Gluconacetobacter	0	0.0%	0	0.0%	0	0.0%	0	0.0%	0	0.0%	0	0.0%	0	0.0%	1	0.0%
Gluconobacter	0	0.0%	0	0.0%	0	0.0%	0	0.0%	0	0.0%	0	0.0%	1	0.0%	0	0.0%
Gordonia	0	0.0%	0	0.0%	0	0.0%	0	0.0%	0	0.0%	0	0.0%	5	0.0%	11	0.0%
Gracilibacter	1	0.0%	0	0.0%	1	0.0%	2	0.0%	0	0.0%	1	0.0%	3	0.0%	1	0.0%
Gracillibacillus	0	0.0%	0	0.0%	0	0.0%	0	0.0%	0	0.0%	5	0.0%	1	0.0%	0	0.0%
Granulicatella	1	0.0%	1	0.0%	0	0.0%	0	0.0%	0	0.0%	1	0.0%	0	0.0%	0	0.0%
Guggenheimella	852	0.2%	39	0.0%	0	0.0%	0	0.0%	4	0.0%	1	0.0%	0	0.0%	1	0.0%
Haemophilus	32	0.0%	8	0.0%	0	0.0%	0	0.0%	0	0.0%	1	0.0%	3	0.0%	1	0.0%
Halanaerobium	0	0.0%	0	0.0%	0	0.0%	0	0.0%	0	0.0%	0	0.0%	2	0.0%	2	0.0%
Haliangium	0	0.0%	0	0.0%	0	0.0%	0	0.0%	0	0.0%	0	0.0%	7	0.0%	3	0.0%
Halomonas	1	0.0%	0	0.0%	0	0.0%	0	0.0%	0	0.0%	0	0.0%	3	0.0%	5	0.0%
Halothiobacillus	1	0.0%	2	0.0%	0	0.0%	0	0.0%	2	0.0%	1	0.0%	2	0.0%	0	0.0%
Helcococcus	23	0.0%	250	0.1%	0	0.0%	0	0.0%	0	0.0%	1	0.0%	0	0.0%	0	0.0%
Helicobacter	0	0.0%	3	0.0%	0	0.0%	0	0.0%	0	0.0%	0	0.0%	1	0.0%	1	0.0%
Heliobacterium	0	0.0%	2	0.0%	2	0.0%	0	0.0%	2	0.0%	6	0.0%	4	0.0%	5	0.0%
Herbaspirillum	1	0.0%	0	0.0%	0	0.0%	0	0.0%	1	0.0%	0	0.0%	2	0.0%	4	0.0%
Herminiimonas	0	0.0%	0	0.0%	0	0.0%	0	0.0%	0	0.0%	2	0.0%	0	0.0%	0	0.0%

hgcl_clade	0	0.0%	0	0.0%	0	0.0%	0	0.0%	0	0.0%	2	0.0%	18	0.0%	25	0.0%
Holophaga	2	0.0%	0	0.0%	0	0.0%	0	0.0%	0	0.0%	0	0.0%	0	0.0%	1	0.0%
Howardella	71	0.0%	86	0.0%	33	0.0%	12	0.0%	40	0.0%	19	0.0%	86	0.0%	108	0.0%
Humicoccus	0	0.0%	0	0.0%	0	0.0%	0	0.0%	1	0.0%	0	0.0%	1	0.0%	0	0.0%
Hydrogenoanaerobacterium	64	0.0%	8	0.0%	55	0.0%	49	0.0%	91	0.0%	41	0.0%	196	0.0%	319	0.1%
Hydrogenophaga	0	0.0%	0	0.0%	0	0.0%	0	0.0%	0	0.0%	0	0.0%	0	0.0%	1	0.0%
Hydrogenophilus	0	0.0%	0	0.0%	0	0.0%	0	0.0%	0	0.0%	0	0.0%	0	0.0%	1	0.0%
Hymenobacter	0	0.0%	0	0.0%	0	0.0%	0	0.0%	1	0.0%	2	0.0%	3	0.0%	2	0.0%
Hyphomonas	0	0.0%	0	0.0%	0	0.0%	0	0.0%	0	0.0%	0	0.0%	0	0.0%	1	0.0%
Hyphomonas	0	0.0%	0	0.0%	0	0.0%	0	0.0%	0	0.0%	0	0.0%	0	0.0%	1	0.0%
Iamia	3	0.0%	0	0.0%	0	0.0%	0	0.0%	0	0.0%	1	0.0%	0	0.0%	1	0.0%
Ideonella	0	0.0%	0	0.0%	0	0.0%	0	0.0%	0	0.0%	0	0.0%	2	0.0%	0	0.0%
Idiomarina	1	0.0%	0	0.0%	0	0.0%	0	0.0%	0	0.0%	0	0.0%	0	0.0%	0	0.0%
Ilumatobacter	0	0.0%	0	0.0%	0	0.0%	0	0.0%	0	0.0%	0	0.0%	1	0.0%	0	0.0%
Incertae_Sedis	11671	2.2%	18307	4.3%	28618	6.4%	19744	3.6%	3978	1.6%	4366	1.7%	15301	3.2%	21718	4.1%
Inhella	0	0.0%	0	0.0%	1	0.0%	1	0.0%	2529	1.0%	5119	2.0%	3	0.0%	2	0.0%
Inquilinus	0	0.0%	0	0.0%	0	0.0%	0	0.0%	280	0.1%	315	0.1%	35	0.0%	8	0.0%
Insolitispirillum	0	0.0%	0	0.0%	0	0.0%	0	0.0%	20	0.0%	1	0.0%	1	0.0%	3	0.0%
Isobaculum	19	0.0%	17	0.0%	0	0.0%	1	0.0%	3	0.0%	3	0.0%	8	0.0%	19	0.0%
Isoptericola	0	0.0%	0	0.0%	0	0.0%	0	0.0%	0	0.0%	0	0.0%	1	0.0%	0	0.0%
Isosphaera	0	0.0%	0	0.0%	0	0.0%	0	0.0%	4	0.0%	1	0.0%	2	0.0%	2	0.0%
Janibacter	0	0.0%	0	0.0%	0	0.0%	0	0.0%	1	0.0%	1	0.0%	0	0.0%	4	0.0%
Janthinobacterium	0	0.0%	0	0.0%	0	0.0%	0	0.0%	0	0.0%	1	0.0%	0	0.0%	0	0.0%
Jeotgalibacillus	0	0.0%	1	0.0%	0	0.0%	0	0.0%	2	0.0%	1	0.0%	0	0.0%	0	0.0%
Johnsonella	58	0.0%	116	0.0%	104	0.0%	53	0.0%	241	0.1%	164	0.1%	1835	0.4%	1906	0.4%
Jonesia	0	0.0%	0	0.0%	0	0.0%	0	0.0%	1	0.0%	0	0.0%	1	0.0%	0	0.0%
Jonquetella	1	0.0%	1	0.0%	0	0.0%	1	0.0%	0	0.0%	3	0.0%	9	0.0%	9	0.0%
Joostella	0	0.0%	0	0.0%	0	0.0%	0	0.0%	1	0.0%	0	0.0%	0	0.0%	0	0.0%
Kineococcus	1	0.0%	0	0.0%	0	0.0%	0	0.0%	2	0.0%	4	0.0%	4	0.0%	12	0.0%
Kineosphaera	0	0.0%	0	0.0%	0	0.0%	0	0.0%	0	0.0%	0	0.0%	1	0.0%	0	0.0%
Kineosporia	0	0.0%	0	0.0%	0	0.0%	0	0.0%	0	0.0%	0	0.0%	3	0.0%	3	0.0%
Kingella	16	0.0%	18	0.0%	0	0.0%	0	0.0%	0	0.0%	0	0.0%	0	0.0%	1	0.0%
Kitasatospora	0	0.0%	0	0.0%	0	0.0%	0	0.0%	2	0.0%	4	0.0%	6	0.0%	9	0.0%

Klugiella	0	0.0%	0	0.0%	0	0.0%	0	0.0%	0	0.0%	1	0.0%	1	0.0%	0	0.0%
Kordia	1	0.0%	1	0.0%	0	0.0%	0	0.0%	0	0.0%	0	0.0%	1	0.0%	0	0.0%
Kordiimonas	0	0.0%	0	0.0%	0	0.0%	0	0.0%	0	0.0%	0	0.0%	1	0.0%	2	0.0%
Kurthia	0	0.0%	0	0.0%	0	0.0%	1	0.0%	0	0.0%	9	0.0%	2	0.0%	4	0.0%
Labrenzia	0	0.0%	0	0.0%	0	0.0%	0	0.0%	3	0.0%	9	0.0%	5	0.0%	3	0.0%
Labrys	0	0.0%	0	0.0%	0	0.0%	0	0.0%	0	0.0%	0	0.0%	0	0.0%	2	0.0%
Laceyella	5	0.0%	0	0.0%	0	0.0%	0	0.0%	2	0.0%	1	0.0%	0	0.0%	0	0.0%
Lachnospira	4	0.0%	1	0.0%	9	0.0%	18	0.0%	1	0.0%	23	0.0%	111	0.0%	233	0.0%
Lacibacter	0	0.0%	0	0.0%	0	0.0%	0	0.0%	0	0.0%	0	0.0%	0	0.0%	2	0.0%
Lactobacillus	9421	1.8%	16942	4.0%	46	0.0%	3	0.0%	56	0.0%	79	0.0%	54	0.0%	54	0.0%
Lactococcus	4	0.0%	24	0.0%	0	0.0%	0	0.0%	89	0.0%	1	0.0%	2	0.0%	1	0.0%
Lawsonia	0	0.0%	0	0.0%	0	0.0%	0	0.0%	0	0.0%	0	0.0%	7	0.0%	10	0.0%
Lechevalieria	0	0.0%	0	0.0%	0	0.0%	0	0.0%	1	0.0%	0	0.0%	0	0.0%	0	0.0%
Leeuwenhoekiella	0	0.0%	0	0.0%	0	0.0%	0	0.0%	0	0.0%	0	0.0%	0	0.0%	3	0.0%
Legionella	1	0.0%	0	0.0%	0	0.0%	0	0.0%	2	0.0%	2	0.0%	41	0.0%	48	0.0%
Leifsonia	1	0.0%	0	0.0%	1	0.0%	0	0.0%	5	0.0%	10	0.0%	17	0.0%	19	0.0%
Lentibacillus	0	0.0%	0	0.0%	0	0.0%	1	0.0%	0	0.0%	0	0.0%	0	0.0%	0	0.0%
Lentzea	0	0.0%	0	0.0%	7	0.0%	0	0.0%	0	0.0%	1	0.0%	0	0.0%	1	0.0%
Leptolinea	0	0.0%	1	0.0%	0	0.0%	0	0.0%	0	0.0%	0	0.0%	3	0.0%	0	0.0%
Leptospira	3	0.0%	1	0.0%	0	0.0%	1	0.0%	3	0.0%	1	0.0%	5	0.0%	5	0.0%
Leptothrix	0	0.0%	0	0.0%	0	0.0%	0	0.0%	0	0.0%	0	0.0%	0	0.0%	1	0.0%
Leptotrichia	33	0.0%	82	0.0%	0	0.0%	0	0.0%	2	0.0%	1	0.0%	0	0.0%	0	0.0%
Leucobacter	0	0.0%	1	0.0%	0	0.0%	0	0.0%	0	0.0%	0	0.0%	2	0.0%	2	0.0%
Leuconostoc	5	0.0%	2	0.0%	0	0.0%	0	0.0%	0	0.0%	0	0.0%	0	0.0%	0	0.0%
Levilinea	1	0.0%	0	0.0%	0	0.0%	0	0.0%	0	0.0%	0	0.0%	3	0.0%	1	0.0%
Limibacter	0	0.0%	1	0.0%	0	0.0%	1	0.0%	1	0.0%	2	0.0%	11	0.0%	14	0.0%
Lishizhenia	1	0.0%	0	0.0%	0	0.0%	0	0.0%	0	0.0%	0	0.0%	2	0.0%	2	0.0%
Longilinea	0	0.0%	0	0.0%	0	0.0%	0	0.0%	0	0.0%	0	0.0%	1	0.0%	0	0.0%
Luteolibacter	0	0.0%	0	0.0%	1	0.0%	0	0.0%	0	0.0%	0	0.0%	0	0.0%	0	0.0%
Lutibacter	0	0.0%	0	0.0%	0	0.0%	0	0.0%	0	0.0%	0	0.0%	1	0.0%	0	0.0%
Lutimonas	0	0.0%	0	0.0%	0	0.0%	0	0.0%	0	0.0%	0	0.0%	0	0.0%	1	0.0%
Lutispora	15	0.0%	3	0.0%	2	0.0%	2	0.0%	12	0.0%	14	0.0%	131	0.0%	94	0.0%
Lysinibacillus	0	0.0%	0	0.0%	0	0.0%	0	0.0%	0	0.0%	0	0.0%	0	0.0%	1	0.0%
Macrococcus	3	0.0%	0	0.0%	2	0.0%	2	0.0%	0	0.0%	3	0.0%	1	0.0%	0	0.0%

Magnetospirillum	0	0.0%	0	0.0%	0	0.0%	0	0.0%	0	0.0%	0	0.0%	25	0.0%	16	0.0%
Mahella	1	0.0%	1	0.0%	1	0.0%	0	0.0%	1	0.0%	0	0.0%	3	0.0%	2	0.0%
Malonomas	0	0.0%	0	0.0%	0	0.0%	0	0.0%	0	0.0%	1	0.0%	2	0.0%	3	0.0%
Mannheimia	854	0.2%	918	0.2%	3	0.0%	3	0.0%	2	0.0%	6	0.0%	6	0.0%	2	0.0%
Maribacter	0	0.0%	0	0.0%	0	0.0%	0	0.0%	0	0.0%	0	0.0%	1	0.0%	0	0.0%
Maricaulis	0	0.0%	0	0.0%	0	0.0%	0	0.0%	1	0.0%	0	0.0%	2	0.0%	0	0.0%
marine_benthic_group	0	0.0%	0	0.0%	0	0.0%	0	0.0%	1	0.0%	0	0.0%	0	0.0%	0	0.0%
marine_group	0	0.0%	0	0.0%	2	0.0%	0	0.0%	5	0.0%	2	0.0%	71	0.0%	12	0.0%
Marinilactibacillus	0	0.0%	0	0.0%	0	0.0%	0	0.0%	0	0.0%	0	0.0%	0	0.0%	1	0.0%
Marinimicrobium	0	0.0%	0	0.0%	0	0.0%	0	0.0%	0	0.0%	1	0.0%	0	0.0%	2	0.0%
Marinitoga	0	0.0%	0	0.0%	0	0.0%	0	0.0%	0	0.0%	0	0.0%	1	0.0%	0	0.0%
Marinobacter	6	0.0%	1	0.0%	0	0.0%	0	0.0%	0	0.0%	0	0.0%	0	0.0%	0	0.0%
Marinobacterium	0	0.0%	0	0.0%	0	0.0%	0	0.0%	0	0.0%	0	0.0%	1	0.0%	0	0.0%
Marmoricola	0	0.0%	0	0.0%	0	0.0%	0	0.0%	2	0.0%	1	0.0%	1	0.0%	1	0.0%
Massilia	0	0.0%	0	0.0%	0	0.0%	0	0.0%	0	0.0%	1	0.0%	8	0.0%	3	0.0%
Megamonas	0	0.0%	1	0.0%	2	0.0%	0	0.0%	5	0.0%	5	0.0%	82	0.0%	79	0.0%
Megasphaera	30	0.0%	5	0.0%	319	0.1%	12	0.0%	28	0.0%	22	0.0%	289	0.1%	294	0.1%
Mesoplasma	0	0.0%	0	0.0%	0	0.0%	0	0.0%	2	0.0%	2	0.0%	0	0.0%	0	0.0%
Mesorhizobium	1	0.0%	1	0.0%	0	0.0%	0	0.0%	0	0.0%	10	0.0%	3	0.0%	6	0.0%
Methylibium	0	0.0%	0	0.0%	1	0.0%	0	0.0%	0	0.0%	3	0.0%	1	0.0%	2	0.0%
Methylobacillus	1	0.0%	0	0.0%	0	0.0%	0	0.0%	0	0.0%	0	0.0%	0	0.0%	0	0.0%
Methylobacter	1	0.0%	1	0.0%	0	0.0%	0	0.0%	0	0.0%	0	0.0%	0	0.0%	0	0.0%
Methylobacterium	3	0.0%	0	0.0%	1	0.0%	1	0.0%	1	0.0%	7	0.0%	15	0.0%	32	0.0%
Methylocaldum	0	0.0%	0	0.0%	0	0.0%	0	0.0%	0	0.0%	0	0.0%	5	0.0%	9	0.0%
Methylocella	0	0.0%	1	0.0%	0	0.0%	0	0.0%	0	0.0%	2	0.0%	4	0.0%	0	0.0%
Methylococcus	2	0.0%	0	0.0%	0	0.0%	0	0.0%	0	0.0%	0	0.0%	0	0.0%	0	0.0%
Methylocystis	0	0.0%	3	0.0%	0	0.0%	0	0.0%	0	0.0%	1	0.0%	15	0.0%	5	0.0%
Methylomonas	40	0.0%	14	0.0%	0	0.0%	0	0.0%	1	0.0%	0	0.0%	0	0.0%	0	0.0%
Methylophaga	3	0.0%	1	0.0%	1	0.0%	0	0.0%	0	0.0%	0	0.0%	0	0.0%	0	0.0%
Methylophilus	0	0.0%	0	0.0%	0	0.0%	0	0.0%	0	0.0%	1	0.0%	0	0.0%	0	0.0%
Methylosarcina	2	0.0%	0	0.0%	0	0.0%	0	0.0%	0	0.0%	0	0.0%	0	0.0%	0	0.0%
Methylosinus	0	0.0%	0	0.0%	0	0.0%	0	0.0%	0	0.0%	0	0.0%	1	0.0%	4	0.0%
Microbacterium	0	0.0%	1	0.0%	0	0.0%	1	0.0%	2	0.0%	0	0.0%	9	0.0%	10	0.0%
Microbulbifer	0	0.0%	0	0.0%	1	0.0%	0	0.0%	2	0.0%	1	0.0%	2	0.0%	3	0.0%

Microcella	0	0.0%	1	0.0%	0	0.0%	1	0.0%	1	0.0%	1	0.0%	3	0.0%	1	0.0%
Micrococcus	0	0.0%	0	0.0%	0	0.0%	0	0.0%	0	0.0%	1	0.0%	0	0.0%	0	0.0%
Microlunatus	0	0.0%	0	0.0%	0	0.0%	0	0.0%	0	0.0%	1	0.0%	360	0.1%	8	0.0%
Micropruina	0	0.0%	0	0.0%	0	0.0%	0	0.0%	0	0.0%	1	0.0%	0	0.0%	1	0.0%
Microscilla	0	0.0%	0	0.0%	0	0.0%	0	0.0%	0	0.0%	0	0.0%	9	0.0%	11	0.0%
Mitsuokella	0	0.0%	0	0.0%	1	0.0%	2	0.0%	9	0.0%	9	0.0%	822	0.2%	426	0.1%
Mogibacterium	317	0.1%	1787	0.4%	278	0.1%	184	0.0%	210	0.1%	169	0.1%	133	0.0%	205	0.0%
Moorella	1	0.0%	1	0.0%	0	0.0%	1	0.0%	1	0.0%	0	0.0%	1	0.0%	1	0.0%
Moraxella	624	0.1%	547	0.1%	4	0.0%	3	0.0%	7	0.0%	3	0.0%	1	0.0%	1	0.0%
Moryella	92	0.0%	139	0.0%	792	0.2%	1134	0.2%	600	0.2%	546	0.2%	1002	0.2%	1332	0.3%
Mucilaginibacter	0	0.0%	0	0.0%	0	0.0%	1	0.0%	2	0.0%	15	0.0%	15	0.0%	16	0.0%
Mucispirillum	0	0.0%	0	0.0%	0	0.0%	0	0.0%	0	0.0%	0	0.0%	3	0.0%	2	0.0%
Muricauda	0	0.0%	1	0.0%	0	0.0%	0	0.0%	0	0.0%	0	0.0%	2	0.0%	2	0.0%
Mycobacterium	0	0.0%	0	0.0%	1	0.0%	0	0.0%	11	0.0%	15	0.0%	88	0.0%	39	0.0%
Mycoplasma	0	0.0%	5	0.0%	0	0.0%	0	0.0%	0	0.0%	1	0.0%	1	0.0%	0	0.0%
Myroides	1	0.0%	0	0.0%	0	0.0%	0	0.0%	2	0.0%	0	0.0%	3	0.0%	1	0.0%
Nannocystis	0	0.0%	0	0.0%	0	0.0%	0	0.0%	0	0.0%	0	0.0%	1	0.0%	0	0.0%
Natranaerobius	0	0.0%	0	0.0%	0	0.0%	0	0.0%	0	0.0%	0	0.0%	1	0.0%	0	0.0%
Natronincola	0	0.0%	0	0.0%	0	0.0%	0	0.0%	0	0.0%	1	0.0%	0	0.0%	0	0.0%
Nautilia	0	0.0%	0	0.0%	0	0.0%	0	0.0%	0	0.0%	0	0.0%	2	0.0%	0	0.0%
Neisseria	529	0.1%	75	0.0%	0	0.0%	1	0.0%	2	0.0%	4	0.0%	0	0.0%	0	0.0%
Neptunomonas	1	0.0%	0	0.0%	0	0.0%	1	0.0%	0	0.0%	0	0.0%	0	0.0%	0	0.0%
Nesterenkonia	0	0.0%	0	0.0%	1	0.0%	0	0.0%	0	0.0%	1	0.0%	4	0.0%	3	0.0%
Nitratifractor	0	0.0%	0	0.0%	0	0.0%	0	0.0%	1	0.0%	0	0.0%	2	0.0%	3	0.0%
Nitrobacter	0	0.0%	0	0.0%	0	0.0%	0	0.0%	0	0.0%	0	0.0%	0	0.0%	1	0.0%
Nitrosomonas	0	0.0%	1	0.0%	0	0.0%	0	0.0%	0	0.0%	8	0.0%	6	0.0%	4	0.0%
Nitrosospira	2	0.0%	0	0.0%	0	0.0%	0	0.0%	0	0.0%	0	0.0%	1	0.0%	0	0.0%
Nitrospina	1	0.0%	6	0.0%	0	0.0%	0	0.0%	1	0.0%	0	0.0%	0	0.0%	0	0.0%
Nocardia	0	0.0%	0	0.0%	2	0.0%	1	0.0%	5	0.0%	2	0.0%	2	0.0%	4	0.0%
Nocardioides	0	0.0%	0	0.0%	0	0.0%	1	0.0%	1	0.0%	1	0.0%	1	0.0%	3	0.0%
Nocardiopsis	0	0.0%	0	0.0%	0	0.0%	0	0.0%	0	0.0%	0	0.0%	0	0.0%	1	0.0%
Novosphingobium	1	0.0%	0	0.0%	0	0.0%	0	0.0%	0	0.0%	2	0.0%	21	0.0%	13	0.0%
NS10	0	0.0%	1	0.0%	0	0.0%	0	0.0%	0	0.0%	0	0.0%	0	0.0%	0	0.0%
NS5	0	0.0%	0	0.0%	0	0.0%	0	0.0%	1	0.0%	0	0.0%	2	0.0%	5	0.0%

Nubsella	8	0.0%	1	0.0%	0	0.0%	0	0.0%	0	0.0%	8	0.0%	5	0.0%	22	0.0%
Oceanisphaera	2	0.0%	0	0.0%	0	0.0%	0	0.0%	0	0.0%	1	0.0%	0	0.0%	0	0.0%
Oceanobacillus	0	0.0%	0	0.0%	0	0.0%	4	0.0%	0	0.0%	3	0.0%	1	0.0%	0	0.0%
Oceanobacter	0	0.0%	0	0.0%	0	0.0%	0	0.0%	1	0.0%	0	0.0%	0	0.0%	0	0.0%
Ochrobactrum	0	0.0%	0	0.0%	0	0.0%	0	0.0%	0	0.0%	0	0.0%	1	0.0%	0	0.0%
Odoribacter	71	0.0%	6	0.0%	0	0.0%	0	0.0%	9	0.0%	0	0.0%	32	0.0%	37	0.0%
Olivibacter	1	0.0%	0	0.0%	0	0.0%	0	0.0%	2	0.0%	17	0.0%	22	0.0%	39	0.0%
Olsenella	87	0.0%	134	0.0%	50078	11.2%	57905	10.4%	197	0.1%	180	0.1%	278	0.1%	447	0.1%
OM182	0	0.0%	1	0.0%	0	0.0%	0	0.0%	0	0.0%	0	0.0%	0	0.0%	2	0.0%
OM27	0	0.0%	0	0.0%	0	0.0%	0	0.0%	0	0.0%	0	0.0%	1	0.0%	1	0.0%
OM43	0	0.0%	0	0.0%	0	0.0%	0	0.0%	0	0.0%	0	0.0%	0	0.0%	1	0.0%
Opitutus	0	0.0%	0	0.0%	0	0.0%	0	0.0%	0	0.0%	0	0.0%	2	0.0%	2	0.0%
Oribacterium	147	0.0%	191	0.0%	43384	9.7%	58527	10.5%	542	0.2%	1093	0.4%	365	0.1%	649	0.1%
Orienta	0	0.0%	0	0.0%	0	0.0%	0	0.0%	1	0.0%	0	0.0%	1	0.0%	0	0.0%
Ornithinibacillus	1	0.0%	0	0.0%	0	0.0%	0	0.0%	0	0.0%	1	0.0%	0	0.0%	0	0.0%
Ornithinimicrobium	0	0.0%	0	0.0%	0	0.0%	0	0.0%	1	0.0%	0	0.0%	0	0.0%	0	0.0%
Oryzihumus	0	0.0%	0	0.0%	0	0.0%	0	0.0%	0	0.0%	0	0.0%	1	0.0%	0	0.0%
Oscillibacter	754	0.1%	171	0.0%	35	0.0%	105	0.0%	216	0.1%	131	0.1%	161	0.0%	330	0.1%
Oscillospira	216	0.0%	64	0.0%	7	0.0%	11	0.0%	1635	0.6%	733	0.3%	81	0.0%	128	0.0%
Ottowia	0	0.0%	0	0.0%	0	0.0%	0	0.0%	0	0.0%	0	0.0%	2	0.0%	4	0.0%
Owenweeksia	2	0.0%	1	0.0%	0	0.0%	0	0.0%	2	0.0%	1	0.0%	26	0.0%	27	0.0%
Oxalicibacterium	0	0.0%	0	0.0%	0	0.0%	0	0.0%	1	0.0%	0	0.0%	0	0.0%	2	0.0%
Oxalobacter	2	0.0%	0	0.0%	0	0.0%	0	0.0%	0	0.0%	0	0.0%	0	0.0%	2	0.0%
Oxalophagus	1	0.0%	0	0.0%	1	0.0%	0	0.0%	0	0.0%	4	0.0%	1	0.0%	2	0.0%
Oxobacter	0	0.0%	0	0.0%	0	0.0%	3	0.0%	0	0.0%	0	0.0%	9	0.0%	17	0.0%
Paenibacillus	13	0.0%	58	0.0%	330	0.1%	125	0.0%	47	0.0%	61	0.0%	64	0.0%	61	0.0%
Paludibacter	69	0.0%	97	0.0%	0	0.0%	5	0.0%	12	0.0%	17	0.0%	25	0.0%	27	0.0%
Pandoraea	1	0.0%	0	0.0%	0	0.0%	0	0.0%	0	0.0%	0	0.0%	0	0.0%	0	0.0%
Pannonibacter	0	0.0%	0	0.0%	0	0.0%	0	0.0%	0	0.0%	0	0.0%	1	0.0%	0	0.0%
Pantoea	0	0.0%	0	0.0%	0	0.0%	0	0.0%	0	0.0%	1	0.0%	2	0.0%	0	0.0%
Papillibacter	11	0.0%	10	0.0%	10	0.0%	3	0.0%	9	0.0%	11	0.0%	57	0.0%	82	0.0%
Parabacteroides	323	0.1%	40	0.0%	20	0.0%	36	0.0%	517	0.2%	425	0.2%	365	0.1%	302	0.1%
Paraferrimonas	0	0.0%	0	0.0%	0	0.0%	0	0.0%	0	0.0%	0	0.0%	1	0.0%	0	0.0%
Parapedobacter	1	0.0%	0	0.0%	0	0.0%	0	0.0%	2	0.0%	0	0.0%	7	0.0%	8	0.0%

Parasporobacterium-Sporobacterium	3	0.0%	18	0.0%	120	0.0%	329	0.1%	137	0.1%	98	0.0%	85	0.0%	195	0.0%
Parasutterella	0	0.0%	0	0.0%	0	0.0%	0	0.0%	0	0.0%	0	0.0%	0	0.0%	1	0.0%
Parvibaculum	0	0.0%	0	0.0%	0	0.0%	0	0.0%	0	0.0%	0	0.0%	1	0.0%	0	0.0%
Parvimonas	184	0.0%	136	0.0%	2	0.0%	2	0.0%	7	0.0%	4	0.0%	30890	6.4%	12473	2.4%
Parvularcula	0	0.0%	0	0.0%	0	0.0%	0	0.0%	0	0.0%	0	0.0%	1	0.0%	1	0.0%
Pasteurella	255	0.0%	94	0.0%	0	0.0%	0	0.0%	3	0.0%	7	0.0%	3	0.0%	2	0.0%
Pectobacterium	0	0.0%	0	0.0%	0	0.0%	1	0.0%	0	0.0%	0	0.0%	1	0.0%	0	0.0%
Pediococcus	2	0.0%	2	0.0%	2	0.0%	0	0.0%	0	0.0%	0	0.0%	3	0.0%	5	0.0%
Pedobacter	20	0.0%	7	0.0%	0	0.0%	2	0.0%	33	0.0%	42	0.0%	178	0.0%	445	0.1%
PeHg26	0	0.0%	0	0.0%	2	0.0%	0	0.0%	2	0.0%	2	0.0%	4	0.0%	23	0.0%
Pelagicoccus	0	0.0%	0	0.0%	0	0.0%	0	0.0%	0	0.0%	0	0.0%	3	0.0%	0	0.0%
Pelobacter	0	0.0%	0	0.0%	0	0.0%	16	0.0%	0	0.0%	0	0.0%	1	0.0%	3	0.0%
Pelomonas	0	0.0%	0	0.0%	0	0.0%	0	0.0%	1	0.0%	1	0.0%	0	0.0%	1	0.0%
Pelotomaculum	1	0.0%	3	0.0%	0	0.0%	0	0.0%	2	0.0%	1	0.0%	3	0.0%	2	0.0%
PeM15	0	0.0%	0	0.0%	0	0.0%	0	0.0%	0	0.0%	2	0.0%	1	0.0%	2	0.0%
Peptococcus	29	0.0%	73	0.0%	4	0.0%	0	0.0%	0	0.0%	0	0.0%	1	0.0%	0	0.0%
Peptoniphilus	65	0.0%	10	0.0%	2	0.0%	0	0.0%	6	0.0%	0	0.0%	0	0.0%	0	0.0%
Peptostreptococcus	401	0.1%	78	0.0%	5	0.0%	1	0.0%	15	0.0%	15	0.0%	7	0.0%	20	0.0%
Peredibacter	1	0.0%	2	0.0%	0	0.0%	0	0.0%	2	0.0%	1	0.0%	2	0.0%	0	0.0%
Perexilibacter	0	0.0%	0	0.0%	0	0.0%	0	0.0%	0	0.0%	0	0.0%	1	0.0%	0	0.0%
Perlucidibaca	1	0.0%	0	0.0%	0	0.0%	0	0.0%	0	0.0%	0	0.0%	7	0.0%	6	0.0%
Persicobacter	2	0.0%	1	0.0%	0	0.0%	0	0.0%	0	0.0%	0	0.0%	0	0.0%	1	0.0%
Petrimonas	112	0.0%	33	0.0%	0	0.0%	0	0.0%	1	0.0%	3	0.0%	0	0.0%	0	0.0%
Petrotoga	0	0.0%	1	0.0%	0	0.0%	0	0.0%	0	0.0%	0	0.0%	0	0.0%	0	0.0%
Phascolarctobacterium	1	0.0%	0	0.0%	0	0.0%	0	0.0%	0	0.0%	0	0.0%	2	0.0%	11	0.0%
Phaselicystis	0	0.0%	0	0.0%	0	0.0%	0	0.0%	0	0.0%	0	0.0%	1	0.0%	0	0.0%
Phenylobacterium	0	0.0%	0	0.0%	0	0.0%	0	0.0%	0	0.0%	0	0.0%	1	0.0%	0	0.0%
Photobacterium	1	0.0%	0	0.0%	0	0.0%	0	0.0%	0	0.0%	0	0.0%	0	0.0%	0	0.0%
Photorhabdus	0	0.0%	0	0.0%	0	0.0%	0	0.0%	0	0.0%	0	0.0%	1	0.0%	0	0.0%
Phyllobacterium	1	0.0%	0	0.0%	0	0.0%	0	0.0%	0	0.0%	0	0.0%	0	0.0%	2	0.0%
Pigmentiphaga	0	0.0%	0	0.0%	0	0.0%	0	0.0%	0	0.0%	0	0.0%	1	0.0%	0	0.0%
Pirellula	0	0.0%	0	0.0%	0	0.0%	0	0.0%	1	0.0%	0	0.0%	4	0.0%	68	0.0%
Planctomyces	0	0.0%	0	0.0%	0	0.0%	1	0.0%	13	0.0%	0	0.0%	5	0.0%	15	0.0%

Planifilum	0	0.0%	0	0.0%	0	0.0%	0	0.0%	1	0.0%	0	0.0%	0	0.0%	0	0.0%
Planococcus	1	0.0%	0	0.0%	0	0.0%	0	0.0%	0	0.0%	2	0.0%	1	0.0%	0	0.0%
Plantibacter	0	0.0%	1	0.0%	0	0.0%	0	0.0%	1	0.0%	0	0.0%	3	0.0%	3	0.0%
Pleomorphomonas	0	0.0%	0	0.0%	0	0.0%	0	0.0%	0	0.0%	0	0.0%	7	0.0%	10	0.0%
Polyangiaceae	0	0.0%	2	0.0%	0	0.0%	0	0.0%	0	0.0%	0	0.0%	0	0.0%	0	0.0%
Polynucleobacter	0	0.0%	0	0.0%	0	0.0%	0	0.0%	1	0.0%	1	0.0%	3	0.0%	2	0.0%
Pontibacter	0	0.0%	0	0.0%	0	0.0%	0	0.0%	0	0.0%	1	0.0%	0	0.0%	0	0.0%
Porphyromonas	4350	0.8%	1831	0.4%	2	0.0%	3	0.0%	56	0.0%	38	0.0%	50	0.0%	51	0.0%
Prevotella	176179	33.3%	58332	13.7%	158345	35.4%	211790	38.2%	106732	42.4%	107257	42.9%	154047	31.9%	172201	32.9%
Propionibacterium	0	0.0%	0	0.0%	0	0.0%	0	0.0%	12	0.0%	18	0.0%	66	0.0%	14	0.0%
Propioniferax	0	0.0%	0	0.0%	0	0.0%	0	0.0%	1	0.0%	1	0.0%	2	0.0%	3	0.0%
Propionispora	1	0.0%	1	0.0%	2	0.0%	0	0.0%	0	0.0%	1	0.0%	9	0.0%	11	0.0%
Propionivibrio	58	0.0%	7	0.0%	0	0.0%	0	0.0%	3	0.0%	1	0.0%	3	0.0%	1	0.0%
Proteiniphilum	18	0.0%	4	0.0%	0	0.0%	0	0.0%	0	0.0%	2	0.0%	10	0.0%	23	0.0%
Pseudacidovorax	0	0.0%	0	0.0%	0	0.0%	0	0.0%	0	0.0%	0	0.0%	1	0.0%	0	0.0%
Pseudidiomarina	0	0.0%	0	0.0%	3	0.0%	1	0.0%	1	0.0%	0	0.0%	0	0.0%	1	0.0%
Pseudoalteromonas	0	0.0%	0	0.0%	0	0.0%	0	0.0%	0	0.0%	1	0.0%	1	0.0%	0	0.0%
Pseudoclavibacter	0	0.0%	0	0.0%	0	0.0%	0	0.0%	1	0.0%	0	0.0%	1	0.0%	0	0.0%
Pseudomonas	6	0.0%	17	0.0%	0	0.0%	0	0.0%	7	0.0%	11	0.0%	26	0.0%	21	0.0%
Pseudonocardia	0	0.0%	0	0.0%	0	0.0%	0	0.0%	0	0.0%	0	0.0%	2	0.0%	6	0.0%
Pseudosphingobacterium	0	0.0%	0	0.0%	0	0.0%	0	0.0%	0	0.0%	0	0.0%	9	0.0%	67	0.0%
Pseudospirillum	0	0.0%	1	0.0%	0	0.0%	0	0.0%	0	0.0%	0	0.0%	0	0.0%	2	0.0%
Psychrobacter	1	0.0%	4	0.0%	0	0.0%	0	0.0%	0	0.0%	0	0.0%	0	0.0%	0	0.0%
Psychromonas	0	0.0%	0	0.0%	0	0.0%	2	0.0%	1	0.0%	0	0.0%	1	0.0%	2	0.0%
Pullulanibacillus	4	0.0%	1	0.0%	1	0.0%	0	0.0%	0	0.0%	0	0.0%	1	0.0%	4	0.0%
Pyramidobacter	176	0.0%	53	0.0%	1	0.0%	8	0.0%	20	0.0%	28	0.0%	8	0.0%	12	0.0%
Pyxidicoccus	0	0.0%	0	0.0%	0	0.0%	0	0.0%	0	0.0%	0	0.0%	1	0.0%	0	0.0%
Ralstonia	0	0.0%	0	0.0%	0	0.0%	0	0.0%	1	0.0%	0	0.0%	2	0.0%	1	0.0%
Rathayibacter	0	0.0%	0	0.0%	0	0.0%	0	0.0%	0	0.0%	0	0.0%	1	0.0%	1	0.0%
RC9	3493	0.7%	2067	0.5%	7878	1.8%	3183	0.6%	13850	5.5%	11044	4.4%	33144	6.9%	34306	6.6%
Rhizobium	0	0.0%	0	0.0%	0	0.0%	0	0.0%	1	0.0%	3	0.0%	5	0.0%	10	0.0%
Rhodanobacter	1	0.0%	15	0.0%	0	0.0%	0	0.0%	0	0.0%	1	0.0%	4	0.0%	3	0.0%
Rhodoblastus	0	0.0%	0	0.0%	0	0.0%	0	0.0%	0	0.0%	0	0.0%	20	0.0%	11	0.0%

Rhodocista	0	0.0%	0	0.0%	0	0.0%	0	0.0%	1	0.0%	1	0.0%	3	0.0%	9	0.0%
Rhodococcus	0	0.0%	1	0.0%	1	0.0%	1	0.0%	6	0.0%	1	0.0%	11	0.0%	7	0.0%
Rhodoferax	5	0.0%	1	0.0%	0	0.0%	0	0.0%	0	0.0%	0	0.0%	2	0.0%	2	0.0%
Rhodopirellula	0	0.0%	14	0.0%	0	0.0%	1	0.0%	76	0.0%	4	0.0%	16	0.0%	23	0.0%
Rhodoplanes	0	0.0%	0	0.0%	0	0.0%	0	0.0%	0	0.0%	0	0.0%	14	0.0%	2	0.0%
Rhodopseudomonas	0	0.0%	1	0.0%	0	0.0%	0	0.0%	0	0.0%	1	0.0%	62	0.0%	51	0.0%
Rhodospirillum	1	0.0%	0	0.0%	0	0.0%	0	0.0%	0	0.0%	0	0.0%	0	0.0%	3	0.0%
Rickettsia	0	0.0%	0	0.0%	0	0.0%	0	0.0%	0	0.0%	0	0.0%	9	0.0%	3	0.0%
Riemerella	1	0.0%	1	0.0%	0	0.0%	0	0.0%	0	0.0%	0	0.0%	0	0.0%	0	0.0%
Robinsoniella	2	0.0%	9	0.0%	21	0.0%	2	0.0%	56	0.0%	39	0.0%	64	0.0%	160	0.0%
Roseatales	0	0.0%	0	0.0%	0	0.0%	0	0.0%	0	0.0%	0	0.0%	3	0.0%	8	0.0%
Roseburia	39	0.0%	311	0.1%	769	0.2%	278	0.1%	282	0.1%	466	0.2%	1313	0.3%	2503	0.5%
Roseiflexus	0	0.0%	0	0.0%	0	0.0%	0	0.0%	0	0.0%	0	0.0%	0	0.0%	1	0.0%
Roseomonas	0	0.0%	0	0.0%	1	0.0%	0	0.0%	0	0.0%	1	0.0%	5	0.0%	3	0.0%
Rothia	1	0.0%	1	0.0%	0	0.0%	0	0.0%	0	0.0%	0	0.0%	2	0.0%	2	0.0%
Rubrobacter	0	0.0%	0	0.0%	0	0.0%	0	0.0%	0	0.0%	0	0.0%	1	0.0%	1	0.0%
Ruminococcus	364	0.1%	111	0.0%	10569	2.4%	14218	2.6%	4452	1.8%	2748	1.1%	3266	0.7%	4201	0.8%
Saccharomonospora	0	0.0%	0	0.0%	0	0.0%	0	0.0%	0	0.0%	1	0.0%	0	0.0%	1	0.0%
Saccharopolyspora	0	0.0%	0	0.0%	0	0.0%	1	0.0%	0	0.0%	0	0.0%	0	0.0%	2	0.0%
Saccharospirillum	1	0.0%	0	0.0%	0	0.0%	0	0.0%	0	0.0%	0	0.0%	0	0.0%	0	0.0%
Salimicrobium	0	0.0%	0	0.0%	0	0.0%	0	0.0%	0	0.0%	0	0.0%	1	0.0%	0	0.0%
Salinibacterium	0	0.0%	0	0.0%	0	0.0%	0	0.0%	0	0.0%	0	0.0%	118	0.0%	1	0.0%
Salinicoccus	0	0.0%	0	0.0%	0	0.0%	0	0.0%	1	0.0%	2	0.0%	0	0.0%	1	0.0%
Salinivibrio	1	0.0%	0	0.0%	0	0.0%	0	0.0%	0	0.0%	0	0.0%	0	0.0%	0	0.0%
Sandarakinotalea	9	0.0%	19	0.0%	0	0.0%	2	0.0%	1	0.0%	0	0.0%	0	0.0%	0	0.0%
Sanguibacter	0	0.0%	0	0.0%	0	0.0%	0	0.0%	1	0.0%	3	0.0%	4	0.0%	5	0.0%
SAR92	0	0.0%	0	0.0%	0	0.0%	0	0.0%	0	0.0%	1	0.0%	0	0.0%	0	0.0%
Sarcina	5	0.0%	5	0.0%	3	0.0%	2	0.0%	5	0.0%	13	0.0%	0	0.0%	14	0.0%
Scardovia	0	0.0%	0	0.0%	0	0.0%	0	0.0%	0	0.0%	0	0.0%	0	0.0%	1	0.0%
Schlesneria	3	0.0%	0	0.0%	0	0.0%	1	0.0%	4	0.0%	1	0.0%	7	0.0%	5	0.0%
Schlesneria	0	0.0%	0	0.0%	0	0.0%	1	0.0%	0	0.0%	0	0.0%	0	0.0%	0	0.0%
Schwartzia	11	0.0%	5	0.0%	186	0.0%	365	0.1%	178	0.1%	206	0.1%	1921	0.4%	1761	0.3%
Sedimentibacter	5	0.0%	2	0.0%	2	0.0%	0	0.0%	2	0.0%	0	0.0%	5	0.0%	9	0.0%
Sediminicola	1	0.0%	0	0.0%	0	0.0%	0	0.0%	0	0.0%	1	0.0%	1	0.0%	0	0.0%

Sediminimonas	0	0.0%	0	0.0%	0	0.0%	0	0.0%	0	0.0%	0	0.0%	0	0.0%	1	0.0%
Segniliparus	0	0.0%	0	0.0%	28	0.0%	26	0.0%	5	0.0%	5	0.0%	6	0.0%	10	0.0%
Selenomonas	32	0.0%	19	0.0%	964	0.2%	130	0.0%	1591	0.6%	1031	0.4%	42683	8.8%	29737	5.7%
Shewanella	1	0.0%	0	0.0%	0	0.0%	0	0.0%	0	0.0%	5	0.0%	2	0.0%	4	0.0%
Shimazuella	2	0.0%	0	0.0%	0	0.0%	0	0.0%	0	0.0%	0	0.0%	0	0.0%	0	0.0%
Shuttleworthia	0	0.0%	0	0.0%	3	0.0%	2	0.0%	4	0.0%	10	0.0%	17	0.0%	25	0.0%
Simplicispira	0	0.0%	0	0.0%	0	0.0%	0	0.0%	0	0.0%	0	0.0%	1	0.0%	0	0.0%
Singulisphaera	1	0.0%	0	0.0%	0	0.0%	0	0.0%	0	0.0%	0	0.0%	4	0.0%	5	0.0%
Skermania	0	0.0%	0	0.0%	0	0.0%	0	0.0%	0	0.0%	0	0.0%	0	0.0%	1	0.0%
Slackia	1	0.0%	2	0.0%	11	0.0%	17	0.0%	14	0.0%	16	0.0%	57	0.0%	62	0.0%
Sneathia	1	0.0%	0	0.0%	0	0.0%	1	0.0%	0	0.0%	0	0.0%	0	0.0%	0	0.0%
Sneathiella	2	0.0%	0	0.0%	1	0.0%	0	0.0%	3	0.0%	30	0.0%	27	0.0%	49	0.0%
Sodalis	0	0.0%	0	0.0%	1	0.0%	0	0.0%	0	0.0%	0	0.0%	0	0.0%	0	0.0%
Soehngenia	439	0.1%	27	0.0%	2	0.0%	1	0.0%	1	0.0%	3	0.0%	3	0.0%	3	0.0%
Solirubrobacter	0	0.0%	0	0.0%	0	0.0%	0	0.0%	0	0.0%	0	0.0%	3	0.0%	1	0.0%
Solitalea	1	0.0%	0	0.0%	0	0.0%	0	0.0%	0	0.0%	2	0.0%	0	0.0%	2	0.0%
Solobacterium	825	0.2%	3987	0.9%	946	0.2%	347	0.1%	711	0.3%	7062	2.8%	536	0.1%	809	0.2%
Sorangium	0	0.0%	0	0.0%	0	0.0%	0	0.0%	0	0.0%	0	0.0%	1	0.0%	0	0.0%
Sphingobacterium	3	0.0%	0	0.0%	1	0.0%	0	0.0%	0	0.0%	2	0.0%	22	0.0%	36	0.0%
Sphingobium	4	0.0%	0	0.0%	0	0.0%	0	0.0%	1	0.0%	2	0.0%	10	0.0%	8	0.0%
Sphingomonas	2	0.0%	0	0.0%	0	0.0%	0	0.0%	4	0.0%	6	0.0%	136	0.0%	121	0.0%
Sphingopyxis	0	0.0%	0	0.0%	0	0.0%	0	0.0%	0	0.0%	0	0.0%	7	0.0%	1	0.0%
Sphingosinicella	0	0.0%	0	0.0%	0	0.0%	0	0.0%	0	0.0%	0	0.0%	1	0.0%	1	0.0%
Spirochaeta	6090	1.2%	1763	0.4%	534	0.1%	1361	0.2%	74	0.0%	58	0.0%	61	0.0%	74	0.0%
Spiroplasma	3	0.0%	0	0.0%	0	0.0%	0	0.0%	0	0.0%	1	0.0%	4	0.0%	2	0.0%
Spirosoma	0	0.0%	0	0.0%	0	0.0%	0	0.0%	0	0.0%	1	0.0%	2	0.0%	0	0.0%
Sporacetigenium	2	0.0%	4	0.0%	0	0.0%	0	0.0%	3	0.0%	2	0.0%	1	0.0%	4	0.0%
Sporanaerobacter	2	0.0%	1	0.0%	0	0.0%	0	0.0%	2	0.0%	1	0.0%	1	0.0%	0	0.0%
Sporichthya	0	0.0%	0	0.0%	0	0.0%	0	0.0%	0	0.0%	0	0.0%	1	0.0%	0	0.0%
Sporomusa	3	0.0%	0	0.0%	1	0.0%	0	0.0%	2	0.0%	0	0.0%	6	0.0%	15	0.0%
Sporosarcina	0	0.0%	0	0.0%	0	0.0%	0	0.0%	0	0.0%	7	0.0%	8	0.0%	7	0.0%
Staphylococcus	3	0.0%	0	0.0%	16	0.0%	10	0.0%	1	0.0%	2	0.0%	0	0.0%	1	0.0%
Stappia	0	0.0%	0	0.0%	0	0.0%	0	0.0%	0	0.0%	1	0.0%	2	0.0%	7	0.0%
Stella	0	0.0%	0	0.0%	0	0.0%	0	0.0%	0	0.0%	0	0.0%	1	0.0%	0	0.0%

Stenotrophomonas	0	0.0%	1	0.0%	0	0.0%	0	0.0%	0	0.0%	0	0.0%	2	0.0%	1	0.0%
Stenoxybacter	1	0.0%	0	0.0%	0	0.0%	0	0.0%	0	0.0%	0	0.0%	0	0.0%	1	0.0%
Stigmatella	0	0.0%	0	0.0%	0	0.0%	0	0.0%	0	0.0%	0	0.0%	1	0.0%	0	0.0%
Streptacidiphilus	0	0.0%	0	0.0%	0	0.0%	2	0.0%	0	0.0%	0	0.0%	1	0.0%	2	0.0%
Streptococcus	997	0.2%	42266	9.9%	40	0.0%	10	0.0%	91	0.0%	67	0.0%	16	0.0%	17	0.0%
Streptomyces	1	0.0%	11	0.0%	2	0.0%	2	0.0%	6	0.0%	4	0.0%	24	0.0%	35	0.0%
Streptosporangium	0	0.0%	1	0.0%	0	0.0%	0	0.0%	0	0.0%	0	0.0%	0	0.0%	0	0.0%
Subdoligranulum	1362	0.3%	277	0.1%	190	0.0%	117	0.0%	6	0.0%	5	0.0%	258	0.1%	271	0.1%
Succiniclasticum	193	0.0%	60	0.0%	486	0.1%	1246	0.2%	58	0.0%	76	0.0%	1881	0.4%	1657	0.3%
Succinispira	0	0.0%	0	0.0%	0	0.0%	0	0.0%	0	0.0%	0	0.0%	108	0.0%	47	0.0%
Succinivibrio	1	0.0%	1	0.0%	647	0.1%	15123	2.7%	41	0.0%	40	0.0%	42	0.0%	35	0.0%
Sulfurimonas	0	0.0%	0	0.0%	1	0.0%	0	0.0%	3	0.0%	3	0.0%	2	0.0%	2	0.0%
Sulfurospirillum	4	0.0%	1	0.0%	0	0.0%	0	0.0%	10	0.0%	12	0.0%	235	0.0%	171	0.0%
Sulfurovum	0	0.0%	1	0.0%	1	0.0%	1	0.0%	0	0.0%	2	0.0%	118	0.0%	136	0.0%
Sutterella	3	0.0%	0	0.0%	0	0.0%	2	0.0%	20	0.0%	18	0.0%	4	0.0%	1	0.0%
Suttonella	54	0.0%	78	0.0%	100	0.0%	6	0.0%	2	0.0%	1	0.0%	22	0.0%	12	0.0%
Syntrophococcus	11	0.0%	15	0.0%	92	0.0%	80	0.0%	60	0.0%	59	0.0%	72	0.0%	124	0.0%
Syntrophomonas	27	0.0%	8	0.0%	5	0.0%	4	0.0%	22	0.0%	24	0.0%	46	0.0%	65	0.0%
Syntrophorhabdus	0	0.0%	0	0.0%	0	0.0%	0	0.0%	0	0.0%	0	0.0%	2	0.0%	1	0.0%
Tannerella	771	0.1%	351	0.1%	13	0.0%	40	0.0%	240	0.1%	163	0.1%	267	0.1%	439	0.1%
Teichococcus	0	0.0%	0	0.0%	0	0.0%	0	0.0%	0	0.0%	0	0.0%	1	0.0%	1	0.0%
Telmatospirillum	1	0.0%	0	0.0%	0	0.0%	0	0.0%	0	0.0%	2	0.0%	12	0.0%	10	0.0%
Tenacibaculum	1	0.0%	5	0.0%	0	0.0%	0	0.0%	1	0.0%	3	0.0%	3	0.0%	3	0.0%
Tepidimicrobium	9	0.0%	2	0.0%	0	0.0%	0	0.0%	0	0.0%	3	0.0%	3	0.0%	8	0.0%
Tepidiphilus	1	0.0%	0	0.0%	0	0.0%	0	0.0%	0	0.0%	0	0.0%	1	0.0%	1	0.0%
Teredinibacter	0	0.0%	0	0.0%	0	0.0%	0	0.0%	0	0.0%	0	0.0%	1	0.0%	2	0.0%
Termite planctomycete cluster	0	0.0%	0	0.0%	0	0.0%	0	0.0%	3	0.0%	0	0.0%	0	0.0%	0	0.0%
Termite Treponema cluster	0	0.0%	0	0.0%	0	0.0%	0	0.0%	0	0.0%	1	0.0%	0	0.0%	1	0.0%
Terrabacter	0	0.0%	0	0.0%	0	0.0%	0	0.0%	0	0.0%	0	0.0%	0	0.0%	1	0.0%
Terracoccus	1	0.0%	0	0.0%	0	0.0%	1	0.0%	0	0.0%	1	0.0%	0	0.0%	0	0.0%
Terriglobus	0	0.0%	0	0.0%	0	0.0%	0	0.0%	0	0.0%	0	0.0%	0	0.0%	2	0.0%
Tetragenococcus	0	0.0%	0	0.0%	0	0.0%	0	0.0%	0	0.0%	1	0.0%	51	0.0%	1	0.0%
Tetrasphaera	8	0.0%	10	0.0%	6	0.0%	6	0.0%	4	0.0%	10	0.0%	0	0.0%	34	0.0%

Thalassomonas	0	0.0%	0	0.0%	0	0.0%	0	0.0%	0	0.0%	0	0.0%	15	0.0%	1	0.0%
Thalassospira	0	0.0%	2	0.0%	2	0.0%	0	0.0%	2	0.0%	2	0.0%	1	0.0%	11	0.0%
Thauera	2	0.0%	1	0.0%	0	0.0%	0	0.0%	0	0.0%	0	0.0%	0	0.0%	0	0.0%
Thermanaeromonas	0	0.0%	1	0.0%	0	0.0%	0	0.0%	0	0.0%	0	0.0%	4	0.0%	0	0.0%
Thermanaerovibrio	0	0.0%	1	0.0%	0	0.0%	0	0.0%	0	0.0%	4	0.0%	18	0.0%	0	0.0%
Thermincola	7	0.0%	5	0.0%	3	0.0%	5	0.0%	11	0.0%	15	0.0%	18	0.0%	25	0.0%
Thermoanaerobacter	2	0.0%	12	0.0%	0	0.0%	0	0.0%	1	0.0%	0	0.0%	2	0.0%	28	0.0%
Thermobacillus	0	0.0%	0	0.0%	67	0.0%	44	0.0%	3	0.0%	2	0.0%	1	0.0%	4	0.0%
Thermodesulforhabdus	0	0.0%	1	0.0%	0	0.0%	0	0.0%	0	0.0%	0	0.0%	0	0.0%	0	0.0%
Thermodesulfovibrio	0	0.0%	0	0.0%	0	0.0%	1	0.0%	0	0.0%	1	0.0%	1	0.0%	0	0.0%
Thermoflavimicrobium	3	0.0%	1	0.0%	27	0.0%	1	0.0%	0	0.0%	1	0.0%	0	0.0%	1	0.0%
Thermohalobacter	1	0.0%	0	0.0%	1	0.0%	0	0.0%	1	0.0%	0	0.0%	1	0.0%	2	0.0%
Thermomonas	0	0.0%	0	0.0%	0	0.0%	0	0.0%	0	0.0%	0	0.0%	17	0.0%	1	0.0%
Thermosinus	0	0.0%	0	0.0%	0	0.0%	0	0.0%	0	0.0%	0	0.0%	1	0.0%	3	0.0%
Thermosipho	2	0.0%	7	0.0%	0	0.0%	0	0.0%	0	0.0%	0	0.0%	0	0.0%	0	0.0%
Thermotoga	11	0.0%	111	0.0%	0	0.0%	0	0.0%	2	0.0%	0	0.0%	0	0.0%	0	0.0%
Thioalkalimicrobium	0	0.0%	0	0.0%	0	0.0%	0	0.0%	0	0.0%	0	0.0%	1	0.0%	2	0.0%
Thioalkalivibrio	0	0.0%	0	0.0%	0	0.0%	0	0.0%	0	0.0%	1	0.0%	0	0.0%	1	0.0%
Thiobacillus	1	0.0%	0	0.0%	0	0.0%	0	0.0%	0	0.0%	0	0.0%	0	0.0%	1	0.0%
Thiocystis	0	0.0%	0	0.0%	0	0.0%	0	0.0%	0	0.0%	0	0.0%	0	0.0%	1	0.0%
Thiofaba	2	0.0%	0	0.0%	0	0.0%	1	0.0%	0	0.0%	1	0.0%	0	0.0%	1	0.0%
Thiomicrospira	0	0.0%	0	0.0%	0	0.0%	0	0.0%	0	0.0%	0	0.0%	1	0.0%	1	0.0%
Thioploca	0	0.0%	0	0.0%	0	0.0%	0	0.0%	0	0.0%	1	0.0%	0	0.0%	0	0.0%
Thioreductor	0	0.0%	0	0.0%	0	0.0%	0	0.0%	0	0.0%	0	0.0%	0	0.0%	1	0.0%
Thiothrix	0	0.0%	0	0.0%	0	0.0%	0	0.0%	1	0.0%	0	0.0%	1	0.0%	2	0.0%
Thiovirga	2	0.0%	0	0.0%	0	0.0%	0	0.0%	0	0.0%	0	0.0%	30	0.0%	0	0.0%
Tindallia	8	0.0%	3	0.0%	172	0.0%	7	0.0%	28	0.0%	25	0.0%	2	0.0%	34	0.0%
Tissierella	5	0.0%	1	0.0%	2	0.0%	0	0.0%	1	0.0%	0	0.0%	0	0.0%	0	0.0%
Tistrella	1	0.0%	0	0.0%	0	0.0%	0	0.0%	0	0.0%	0	0.0%	0	0.0%	1	0.0%
Tolumonas	0	0.0%	0	0.0%	5	0.0%	0	0.0%	0	0.0%	0	0.0%	403	0.1%	1	0.0%
Treponema	17	0.0%	8	0.0%	2078	0.5%	3515	0.6%	356	0.1%	221	0.1%	7	0.0%	437	0.1%
Trichococcus	0	0.0%	1	0.0%	0	0.0%	0	0.0%	0	0.0%	0	0.0%	0	0.0%	0	0.0%
Tropheryma	0	0.0%	0	0.0%	0	0.0%	0	0.0%	0	0.0%	1	0.0%	0	0.0%	2	0.0%
Tsukamurella	0	0.0%	0	0.0%	0	0.0%	0	0.0%	0	0.0%	0	0.0%	20	0.0%	1	0.0%

Turicibacter	0	0.0%	0	0.0%	0	0.2%	0	0.1%	0	0.0%	0	0.0%	957	0.0%	0	0.0%
Turicibacter	8	0.0%	3	0.0%	769	0.0%	345	0.0%	17	0.0%	17	0.0%	2	0.2%	23	0.0%
Uliginosibacterium	1	0.0%	0	0.0%	0	0.0%	0	0.0%	0	0.0%	0	0.0%	0	0.0%	0	0.0%
Undibacterium	0	0.0%	2	0.0%	0	0.0%	0	0.0%	0	0.0%	0	0.0%	0	0.0%	2	0.0%
Ureibacillus	1	0.0%	0	0.0%	0	0.0%	0	0.0%	0	0.0%	0	0.0%	0	0.0%	0	0.0%
vadinBC27	31383	5.9%	10919	2.6%	283	0.1%	145	0.0%	614	0.2%	501	0.2%	9	0.0%	1251	0.2%
Vagococcus	1	0.0%	0	0.0%	0	0.0%	0	0.0%	0	0.0%	0	0.0%	0	0.0%	0	0.0%
Variovorax	0	0.0%	1	0.0%	1	0.0%	0	0.0%	2	0.0%	0	0.0%	9	0.0%	5	0.0%
Veillonella	242	0.0%	108	0.0%	45	0.0%	3	0.0%	9	0.0%	3	0.0%	35	0.0%	47	0.0%
Victivallis	17	0.0%	18	0.0%	257	0.1%	124	0.0%	257	0.1%	552	0.2%	107	0.0%	305	0.1%
Virgibacillus	3	0.0%	3	0.0%	0	0.0%	0	0.0%	2	0.0%	4	0.0%	5	0.0%	6	0.0%
Volucribacter	0	0.0%	1	0.0%	0	0.0%	0	0.0%	0	0.0%	0	0.0%	0	0.0%	0	0.0%
Weissella	0	0.0%	0	0.0%	0	0.0%	0	0.0%	0	0.0%	0	0.0%	2	0.0%	0	0.0%
Williamsia	0	0.0%	0	0.0%	0	0.0%	0	0.0%	0	0.0%	0	0.0%	2	0.0%	0	0.0%
Xanthomonas	0	0.0%	0	0.0%	0	0.0%	0	0.0%	1	0.0%	1	0.0%	1	0.0%	2	0.0%
Xylanibacter	65	0.0%	21	0.0%	543	0.1%	291	0.1%	597	0.2%	317	0.1%	1559	0.3%	2700	0.5%
YB-45	0	0.0%	2	0.0%	0	0.0%	0	0.0%	0	0.0%	0	0.0%	0	0.0%	2	0.0%
Yeosuana	0	0.0%	0	0.0%	0	0.0%	0	0.0%	0	0.0%	1	0.0%	0	0.0%	0	0.0%
Zhouia	0	0.0%	0	0.0%	0	0.0%	0	0.0%	0	0.0%	0	0.0%	2	0.0%	4	0.0%
Zimmermanella	0	0.0%	0	0.0%	0	0.0%	0	0.0%	0	0.0%	1	0.0%	1	0.0%	0	0.0%
Zimmermannella	0	0.0%	0	0.0%	0	0.0%	0	0.0%	0	0.0%	0	0.0%	0	0.0%	2	0.0%
Zobellella	0	0.0%	0	0.0%	0	0.0%	0	0.0%	0	0.0%	0	0.0%	0	0.0%	1	0.0%
Zoogloea	3	0.0%	0	0.0%	0	0.0%	0	0.0%	0	0.0%	0	0.0%	0	0.0%	0	0.0%
Zunongwangia	0	0.0%	0	0.0%	0	0.0%	0	0.0%	1	0.0%	0	0.0%	9	0.0%	2	0.0%
